# PilOT-Measure: a mobile 3D depth sensing application to support accurate and efficient clinician-led home-based falls risk assessments

**DOI:** 10.1186/s12911-025-03149-7

**Published:** 2025-09-24

**Authors:** Zear Ibrahim, Arthur G. Money, Anita Atwal, Georgia Spiliotopoulou

**Affiliations:** 1 Department of Computer Science, Brunel University London, Uxbridge, UB8 3PH UK; 2 Department of Childhood and Social Care, University of East London, London, E15 4LZ UK; 3 Department of Clinical Sciences, Brunel University London, Uxbridge, UB8 3PH UK

**Keywords:** Falls prevention, Falls risk factors, Virtual reality, Augmented reality, Depth sensing, Occupational therapy, 3D visualisation, Technology for health, Measurement, 3D mobile application

## Abstract

**Background:**

An aging global population, coupled with high levels of assistive equipment abandonment, has propelled increases in falls-related injuries at home. Equipment abandonment occurs, in-part, due to inaccurate measurements of the patient’s home taken during the falls risk assessment process. There is an urgent need to explore the value of new digital mobile technologies to help clinicians to take more efficient and effective measurements of patient’s home, thereby enhancing the efficacy of falls risk assessments and potentially minimising equipment abandonment.

**Aim:**

The aim of this study is to present and evaluate the accuracy and efficiency of PilOT-Measure, a digital mobile 3D depth-sensor-enabled measurement guidance application for use by clinicians carrying out falls risk assessments.

**Methods:**

Twenty-one trainee and registered Occupational Therapists took part in this repeated-measures, mixed methods study to evaluate measurement accuracy, task completion time, and overall system usability and user perceptions of the application.

**Results:**

For measurement accuracy, PilOT-Measure outperformed current state of the art handheld tape measure and paper-based measurement guidance booklet. For accuracy consistency, the handheld tape measure and booklet was more consistently accurate for six out of 11 cases. However, PilOT-Measure tended to facilitate significantly faster task completion times, suggesting potential task efficiency benefits. In terms of usability, participants favoured PilOT-Measure and saw potential to reduce administrative tasks and support joint decision-making. Concerns about marker placement on reflective surfaces and patient privacy were noted.

**Conclusions:**

This study highlights the positive role that mobile depth-sensing technologies can potentially play in improving the efficiency and accuracy of falls risk assessments, hence, reducing levels of equipment abandonment and falls related injuries at home. Future work will focus on improving marker placement, measurement accuracy, and accuracy consistency and explore the potential of using PilOT-Measure as a falls risk patient self-assessment tool.

**Supplementary Information:**

The online version contains supplementary material available at 10.1186/s12911-025-03149-7.

## Background

There is an ever increasing demand for global healthcare resources, largely as a result of an ageing world population [1, 2]. In the UK, the NHS is facing significant challenges in coping with increased demand for resources due to ever increasing life-expectancies, coupled with increasingly constrained public health resource budgets [3]. Innovation in the use of technology for healthcare is seen as one of the few areas that promise to reduce costs and improve efficiency whilst simultaneously improving the quality of service and healthcare delivery to patients [4]. The UK government is clearly committed to the use of Information and Communication Technology (ICT) in healthcare as a key tool in delivering more efficient, patient-centred, and personalised care [5]. Government initiatives such as ‘Going paperless by 2018’ and the Five Year Forward View [6, 7] have helped catalyse a move towards the adoption of innovative ICT applications that enable a shift away from more traditional paternalistic paper-based models of care, towards ICT based interventions that support more efficient patient-centred interventions that better support clinicians and enable patients to take more responsibility for their own care. Despite these initiatives, there is still much work to do if the full potential of ICT is to be realised across the full range of healthcare settings [6, 8].

Falls prevention research within the field of occupational therapy is by no means exempt from global health resource challenges. As a result of an ageing population, the number of falls related injuries has increased significantly in recent years [9]. In the UK, falls are the most common cause of death from injury in over 65s [10]. The annual cost to the NHS of falls related injuries is currently estimated at £4.4 billion, which is anticipated to continue to rise in coming years [10]. The home living environment poses a significant risk in terms of exposing older adults to falls risk with 30% of older adults over 65, and 50% of adults over 80 who live independently, falling each year [11]. A key fall prevention intervention strategy is to make changes and adaptations to the patient’s home living environment, with the aim of removing existing fall hazards and reducing the future risk of falling. The prescribed home adaptations normally take the form of the fitment of assistive equipment (AE) such as stair handrails, bathroom grab rails, toilet and chair raisers around the patient’s home. Prescription of home adaptations and the fitment of AE within the home are becoming an increasingly important intervention. When prescribed accurately, home adaptations are believed to have the potential to significantly reduce the risk of falling, reducing costs, and also improving quality of life by enabling the patient to age in place and live independently at home for longer [12].

Despite the many potential benefits of prescribing home adaptations and the fitment of AE, almost one third of all assistive equipment that is installed within the home is abandoned by the patient after fitment [13–15]. One of the key reasons for equipment abandonment is due to measurement inaccuracies that occur when manual measurements are taken of key items within the home environment, resulting in the subsequent inaccurate prescription of AE and adaptations within the home setting [12].

### Falls risk assessments and the prescription of assistive equipment

Before any home adaptations can be prescribed, a clinician (typically an occupational therapist) must carry out a falls risk assessment (FRA). This involves the occupational therapist visiting the patient’s home, assessing the living environment, and identifying potential falls risks. There are three key parts to the FRA:

1) Gather information about the patient’s functional abilities.

2) Measure fittings and key items of furniture.

3) Prescribe AEs to be installed within the home based on the information and measurements gathered.

**Fig. 1:**
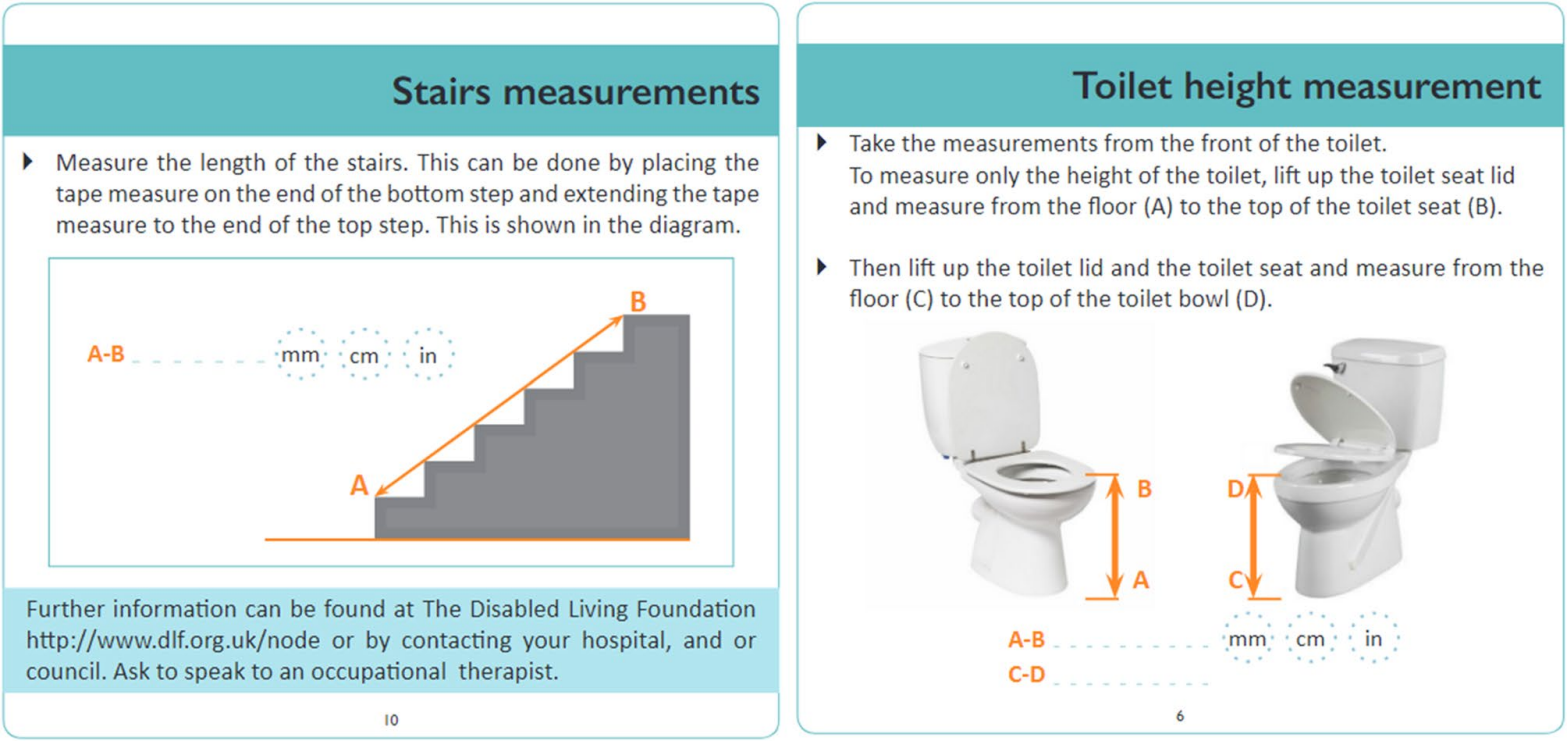
Example stairs (left) and toilet (right) measurement guidance

The two key tools used to carry out FRAs is a hand-held retractable tape measure and a paper-based measurement guidance booklet. The booklet provides measurement guidance instructions for the five items of furniture that are most associated with causing falls within the home: bed, bath, chair, stairs, toilet as well as the popliteal height of the patient. The booklet serves as a guide to help ensure the clinician takes accurate point-to-point [12, 16] measurements of fittings and key furniture items within the home. It also provides space for the clinician to write down each of the measurements that have been taken [13, 17, 18]. The measurement guidance provides 2D illustrations of each item of furniture and includes annotated measurement arrows that are overlaid onto each item of furniture, hence serving as prompts to indicate the precise point-to-point measurements that are required for each of the five furniture items. It is important the that the point-to-point measurement data is accurate as it is primarily used to formulate an assessment and to prescribe the fitment of appropriately sized AE within the home. Figure 1 provides examples of the booklet measurement guidance for stairs and toilet height.

Despite the provision of detailed paper-based measurement guidance, around 30% of all AE that is fitted within the home is abandoned by patients within the first year [13–15]. A key reason for the abandonment of equipment is a ‘poor fit’ between the home environment, the AE, and the person it has been prescribed for [19, 20]. The impact of equipment abandonment is wide-spread and has significant negative effects on the patient, including reduced independence and quality of life, increased exposure to falls risks [21], as well as a depletion of already scarce healthcare resources [22]. Given the significant issues that occur as a result of inaccurate measurements during FRAs, there is a need to identify novel mobile technology-based solutions that provide enhanced support to clinicians whilst carrying out FPAs and enable them to take more reliable and accurate measurements of the home environment. Improved measurement accuracy would enable more appropriate prescription of AE which would ultimately achieve a better fit between the adapted home environment and the patient’s occupational needs, hence reducing levels of equipment abandonment.

### Simulated 3D visualisation technologies to support falls risk assessments

Simulated 3D visualisation involves the use of computer-generated graphics applications that leverage aspects of human visual perception to present images that simulate the representation of three-dimensional (3D) objects in two-dimensional (2D) space. Simulated 3D visualisations often allow the user to interact with on-screen 3D models of objects, providing functions such as object selection, rotation and zoom. The use of 3D objects and visualisation has been identified as having significant potential in overcoming the challenges of existing conventional 2D paper-based clinical tools with the potential to provide the visual quality and detail necessary to conceptualise visual cues as part of a particular treatment and assessment [23]. For example, Jang et al. [24] explored the use of 3D visualisation technologies within the healthcare setting, who enable patients to express their pain symptoms more effectively and accurately to the clinician by annotating specific regions on an on-screen interactive 3D model of the human body. Fall Sensei [25] is an interactive first-person 3D exploration game that allows patients to learn about falls risk factors that may occur within the home. The home environment is modelled in simulated 3D space which the player can explore and can progress through the game by accurately identifying potential falls hazards.

Simulated 3D modelling and visualisation technologies have been identified as having significant potential to improve the quality of measurement guidance for FRAs [26]. A small number of studies have already started to explore the potential of using 3D visualisation technologies to improve measurement accuracy and enhance the level of support offered to clinicians whilst carrying out FRAs. Guidetomeasure-OT [27] is a mobile tablet-based application that uses 3D visualisation technologies specifically designed to support occupational therapists carrying out FRAs. It aims to replace the paper-based measurement guidance booklet with an equivalent digital 3D measurement guidance application which allows the clinician to rotate and zoom into 3D representations of furniture measurement guidance. A separate study presents Guidetomeasure-3D [28] which is also a 3D visualisation tablet-based application, but which was designed to support patients (as opposed to occupational therapists) in the task of carrying self-administered falls risk assessments. Both [27] and [28] produced some promising results when using this technology as a surrogate for paper-based guidance with improvements being reported in task efficiency, usability, and measurement accuracy compared with the 2D paper-based equivalent. Other studies that focus specifically on improving FRAs using 3D visualisation technology include a qualitative study exploring occupational therapists’ perceptions of using 3D visualisation technologies to facilitate the FRA process [29]. This study reported that occupational therapists are positive about embracing new technologies and see numerous potential benefits of using such applications in practice. A study exploring the feasibility of using 3D visualisation home interior design software to assist in the pre-discharge home adaptation process found that occupational therapists were positive about the potential use of such applications to improve collaboration with a number of patient groups [30]. Similarly, an exploratory study considered the potential value of using 3D-MAP, a prototype 3D visualisation application designed for older adult patients in the assistive equipment provision process [31]. The study found that the application was seen to have potential in being deployed in a range of collaborative patient-practitioner settings but that further research was required to evaluate the clinical utility of such an application. Home Quick [32] is an application that was developed to explore the potential of using a range of mobile ICTs deployed on a smart-phone or tablet to enable virtual home-visits to take place. The study found that augmented virtual home visits increased the efficiency of home visits whilst also achieving a similar level of measurement accuracy compared with that achieved when carrying out in-person home-visits using traditional paper-based guidance.

Whilst it is clear from existing research that FRA home visits can feasibly be supported and augmented using mobile 3D visualisation technologies, there is a limit to the accuracy and efficiency gains that these technologies alone can achieve over paper-based equivalents [26]. Despite the promising results reported in [27] and [28], both of these studies recommend that future research directions should explore the use of mobile 3D depth sensing-enabled camera technologies to help further improve measurement accuracy and the support that can be achieved when using mobile technologies to support FRAs. Similarly, a recent literature survey that considered the state of the art in computer mediated reality technologies in healthcare concluded that there is a need to explore the potential value of mobile 3D depth sensing-enabled camera technologies within a wider range of patient-centred care settings [26].

### Mobile depth sensing to augment falls risk assessments

Depth sensors (also referred to as range sensors) provide the capability to capture digital 3D information pertaining to the construction and arrangement of the physical world and the objects within it. Depth sensors are able to scan the local environment and build up detailed digital 3D map representations that could feasibly facilitate the accurate point-to-point measurements of objects that exist within that environment [33]. Whilst numerous depth perception technologies exist, laser-based light detection and ranging (LiDAR) and Infrared (IR) sensors are most commonly used to equip smartphone and tablet devices with 3D depth perception capabilities. LiDAR sensors calculate depth by emitting laser pulses of light to scan the local environment and calculate the time it takes for the beam of light to hit a target and return to the sensor. By carrying out a series of Time of Flight (ToF) calculations, a detailed digital 3D map of the environment can be built up. Due to their relatively low cost, reliability, accuracy, low required computational overhead, and the indoor feasibility of phase difference returning direct distances, LiDAR and Infrared (IR) ToF sensors are a particularly good fit for enabling accurate depth measurement on smartphones and tablet devices. In recent years, ToF sensors are becoming ubiquitously available on some mobile platforms. Many leading mobile phone manufacturers, including Apple and Samsung, have started to include on-board 3D depth cameras using LiDAR and Infrared ToF sensors as standard features on many of their smartphones and tablets [34]. Furthermore, platforms such as the Kinect 1 and 2 [35], Tango [36, 37], Prefab 2, Occipital [38, 39], and Huawei AR engine [40] are well known commercial outlets to which ToF technologies has been integrated. Some benefits of having on-board 3D depth sensor enabled cameras on a smartphone/tablet include significantly enhanced image focusing accuracy, enhanced focus speed, and improved facial recognition. This is due to the detailed 3D depth mapping data and spatially accurate representations that 3D depth sensors are able to generate about the local environment and the size and position of objects within it. The extra level of 3D depth data also provides many new opportunities to deliver significantly enhanced features within augmented reality (AR) applications [41]. In particular, social media platforms such as TikTok and Snapchat have already started to capitalise on the availability of 3D depth data to create faster, more intuitive, and more user-friendly AR filters and lenses that more accurately identify and interact with real-world objects at their actual point of location within 3D space [42].

Some areas of occupational therapy research have already identified the potential value that mobile ‘depth aware’ devices may have in practice. For example, Kaminska et al. [43] use Xbox 360 Kinect depth sensors to deliver virtual reality (VR) interactive falls prevention exercise training in the form of a range of interactive exergames that track and interact with the older adult patient’s movements whilst playing. The study found that depth enabled VR exergaming increases motor training and can help reduce the risk of falling in the long term. Phirom et al. [44] also used Xbox Kinect 360 to deliver game-based training to older adults and found that it was effective in reducing physiological fall risk and helps to improve cognitive function. Yang et al. [45] develop an exergame using Kinect depth sensors to help engage and support older adults carrying out balance training. The results reveal that engaging in depth enabled exercises improve participants’ overall balance ability. Hsieh et al. [46] developed a VR application using Kinect depth sensors to help older-adults better engage in fall prevention balance ability exercises. Improvements were shown in the control group through the results of balance assessment scales. Apart from fall-prevention, depth enabled devices have also been proposed for rehabilitation, assessment, and monitoring systems; for example Dutta, Chugh [47] use Wii depth sensors to capture balance and posture data from patients carrying out grab and reach tasks. Analysis of this data revealed that the Center-of-Pressure (CoP), lean-angle and maximum Center-of-Mass (CoM) correlate significantly with the clinical balance scores (Berg Balance Scale). Similarly, Pu, Sun [48] investigated key factors affecting the balance in older adults using a Kinect where the static and dynamic balance functions were shown to be related. Gama, Chaves [49] proposed a system for post-stroke upper limb rehabilitation and found that the proposed depth sensors are accurate enough for future studies. Stone and Skubic [50] studied gait in five elderly subjects in their home during a 4-month period and proposed a methodology for gait monitoring using a Kinect depth sensor. Kakadiaris, Islam [51] proposed a home anatomy education system using structure sensor to educate prospective patients on surgical procedures.

Although there are examples of studies that explore the value of using depth sensing technologies for fall prevention [52–54], to the best of our knowledge, there is no existing research that builds on the 3D visualisation work of [27] and [28] to explore how the new generation of mobile 3D depth enabled tablet devices can be exploited to further help to support clinicians when carrying out the FRAs. Mobile 3D depth enabled devices have the potential to provide on-screen digital measurement guidance in a similar way that standard non-depth enabled tablet and smartphone devices can. However, they also have the potential to allow clinicians to carry out point-to-point measurements of objects directly on-screen, instead of a handheld tape measure, due to the additional depth mapping information that these devices generate about the objects in the local environment. No existing research has developed a mobile 3D depth enabled measurement guidance application deployed on a tablet device, that seeks to replace the currently used 2D paper-based measurement guidance booklet and handheld tape measure. Such applications have the potential to transform the current state of the art in falls risk assessments by fully digitising the FRA home visit and potentially improving measurement accuracy and the way in which measurement guidance is delivered to the clinician. Therefore, there is a need to develop a mobile 3D depth enabled measurement guidance and on-screen point-to-point measurement application and explore the clinical utility of its performance compared with the state-of-the art handheld tape measure and 2D paper-based equivalent.

### Research aim & questions

The aim of this study is of two-fold. First, to present the PilOT-Measure application, a pilot mobile 3D depth enabled measurement guidance and on-screen point-to-point measurement application developed for use by clinicians carrying out FRAs. PilOT-Measure is deployed on a depth-perception enabled tablet which uses active ToF range sensors and passive-parallax approaches [55]. Second, the aim is to evaluate the performance and explore the clinical utility of the PilOT-Measure application compared with the 2D state of the art paper-based guidance booklet and handheld tape measure equivalent. This is a mixed methods study which aims to establish the relative efficiency and effectiveness of the system in conjunction with its feasibility and perceptions, from a clinician’s perspective, in terms of user satisfaction and attitudes towards adopting and using this new technology in practice. Specifically, the following research questions are addressed as part of this study:

1. Does PilOT-Measure, on average, enable more *accurate* recording of measurements, compared with the handheld tape-measure and paper-based booklet?
2. Does PilOT-Measure enable more *consistently accurate* recording of measurements, compared with the handheld tape-measure and paper-based booklet?
3. Does PilOT-Measure enable measurements to be recorded more *efficiently*, compared with the handheld tape-measure and paper-based booklet?
4. How *satisfied*, in terms of usability, are users of PilOT-Measure, compared with the handheld tape-measure and paper-based booklet?
5. What are the OTs view of PilOT-Measure’s *perceived challenges*, *opportunities*, and their *intention to adopt* this technology in practice?

## The PilOT-Measure digital measurement application

This section presents details about PilOT-Measure, a mobile 3D digital application that has been developed to support OT clinicians in carrying out measurement tasks as part of the FRA procedure. A full application walkthrough is presented in Sect. “Application walkthrough”. The system architecture, and formal presentation of the point-to-point measurement mapping technique developed specifically for PilOT-Measure, is presented in Sect. “System architecture”.

### Application walkthrough

This section provides a walkthrough of the PilOT-Measure application.

#### Launch screen and main menu

**Fig. 2:**
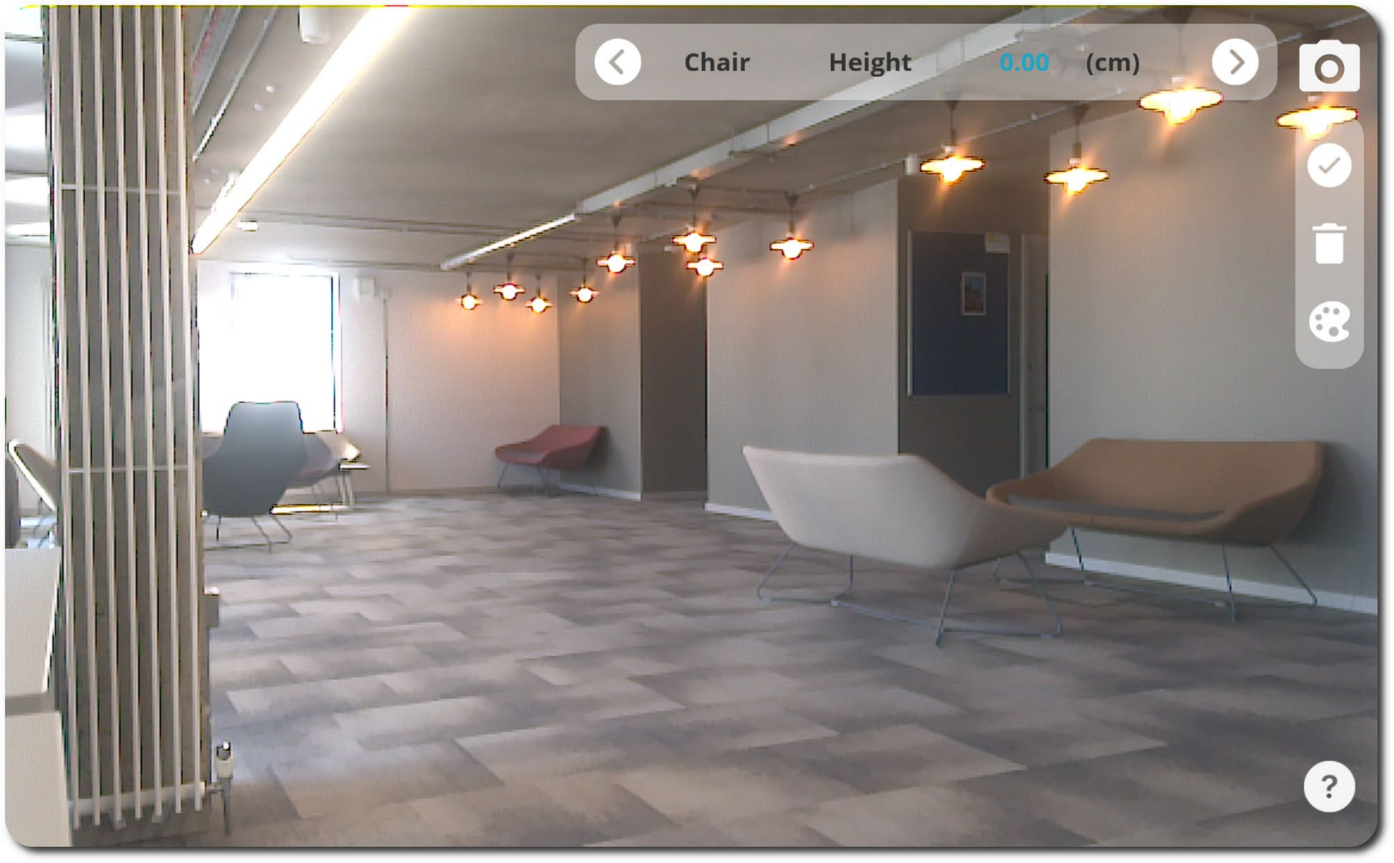
PilOT-Measure launch screen, application control panels (top right), and camera point-of-view

PilOT-Measure was developed in Unity3D, which supports deployment across a range of mobile platforms. It has been designed to be deployed on tablet or smartphone devices that have on-board depth perception capabilities. On launching the application, the first screen that the user is presented with is a direct point-of-view of the of the device’s camera along with the key application control panels which are overlaid on the right-hand side and top right of the screen. Figure 2 presents the PilOT-Measure launch screen.

PilOT-Measure incorporates an unobtrusive General User Interface (GUI) overlay that is always visible irrespective of the device’s POV or positioning in the physical world. Additionally, PilOT-Measure control panel design opts to include no sub-menus, which is in-line with the official iOS and Android material design guidelines and AR-UX standards [56, 57].

#### Onboarding instructions

**Fig. 3:**
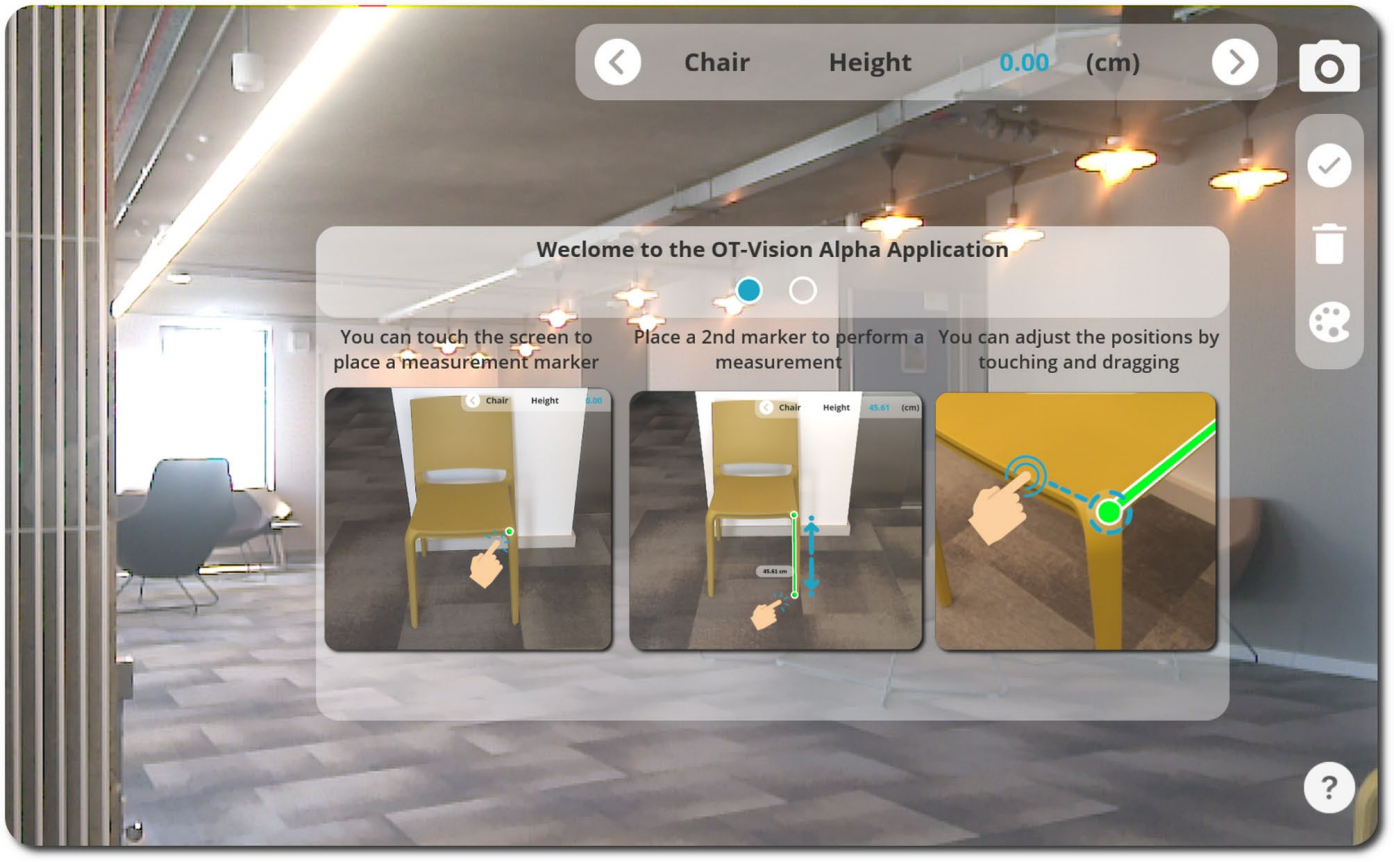
PilOT-Measure onboarding screen 1, taking on-screen point-to-point measurements

**Fig. 4:**
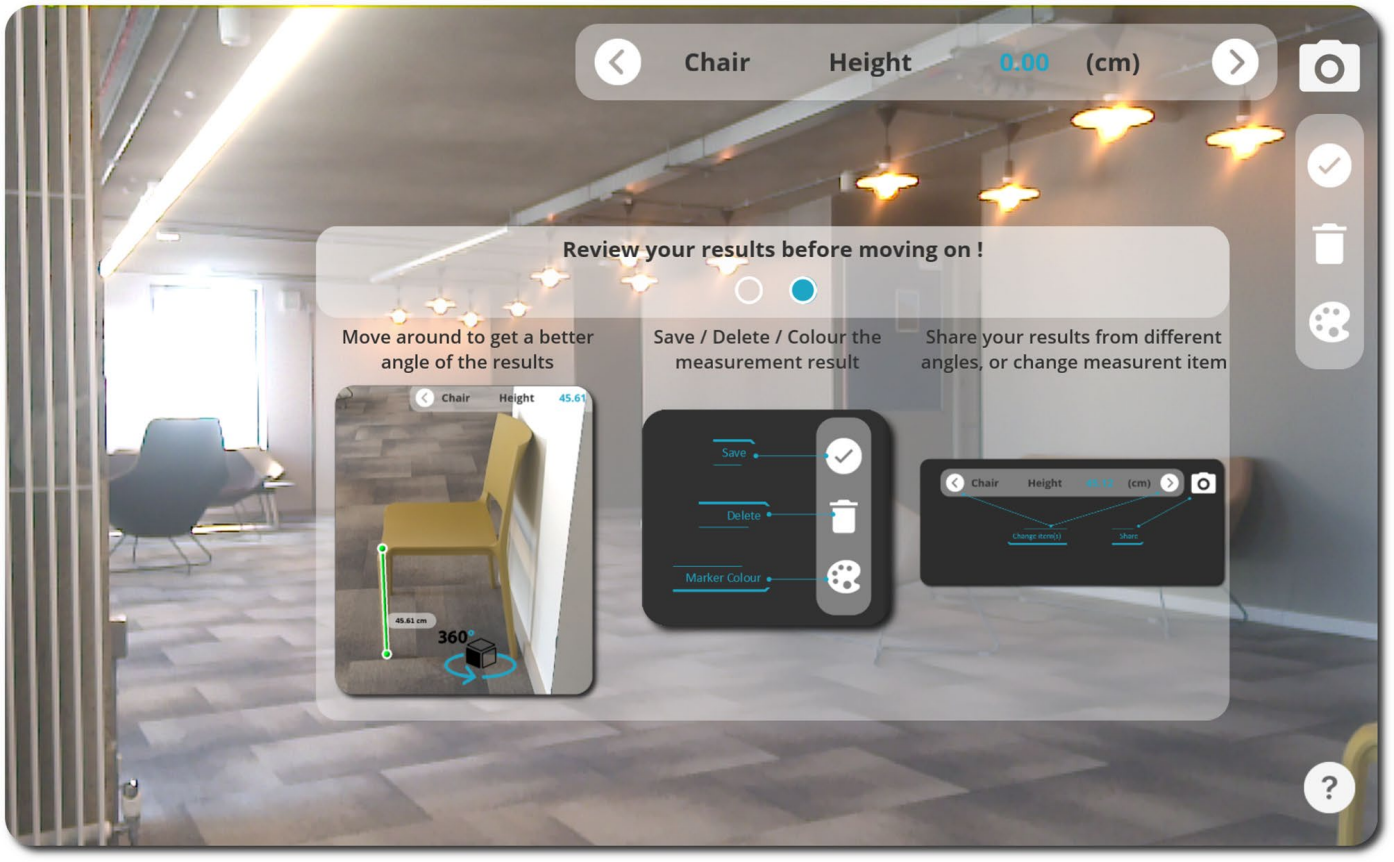
PilOT-Measure onboarding screen 2, adjusting, storing, and communicating measurements results

Users launching the application for the first time can access some key instructional content about how to use the application via the question mark icon in the bottom right corner of the screen. The onboarding instructions enable users to swipe across a set of instruction panels that provide orientation around both UX and GUI elements of the application. The instructions provide detail about how point-to-point measurements can be taken directly on-screen using the point-of-view of the device’s camera output, as shown in Fig. 3. Users are also provided with an overview of how measurements can be deleted, saved, adjusted, and shared, as shown in Fig. 4.

The onboarding screens maintain the direct point-of-view (POV) overlay as a background which provides the context for the point-to-point measurement instructions that are provided in the main dialogue window. According to existing material design guidelines, it is good practice to provide mobile depth sensing instructions in the form of onboarding overlays [56].

#### Taking and recording measurements

**Fig. 5:**
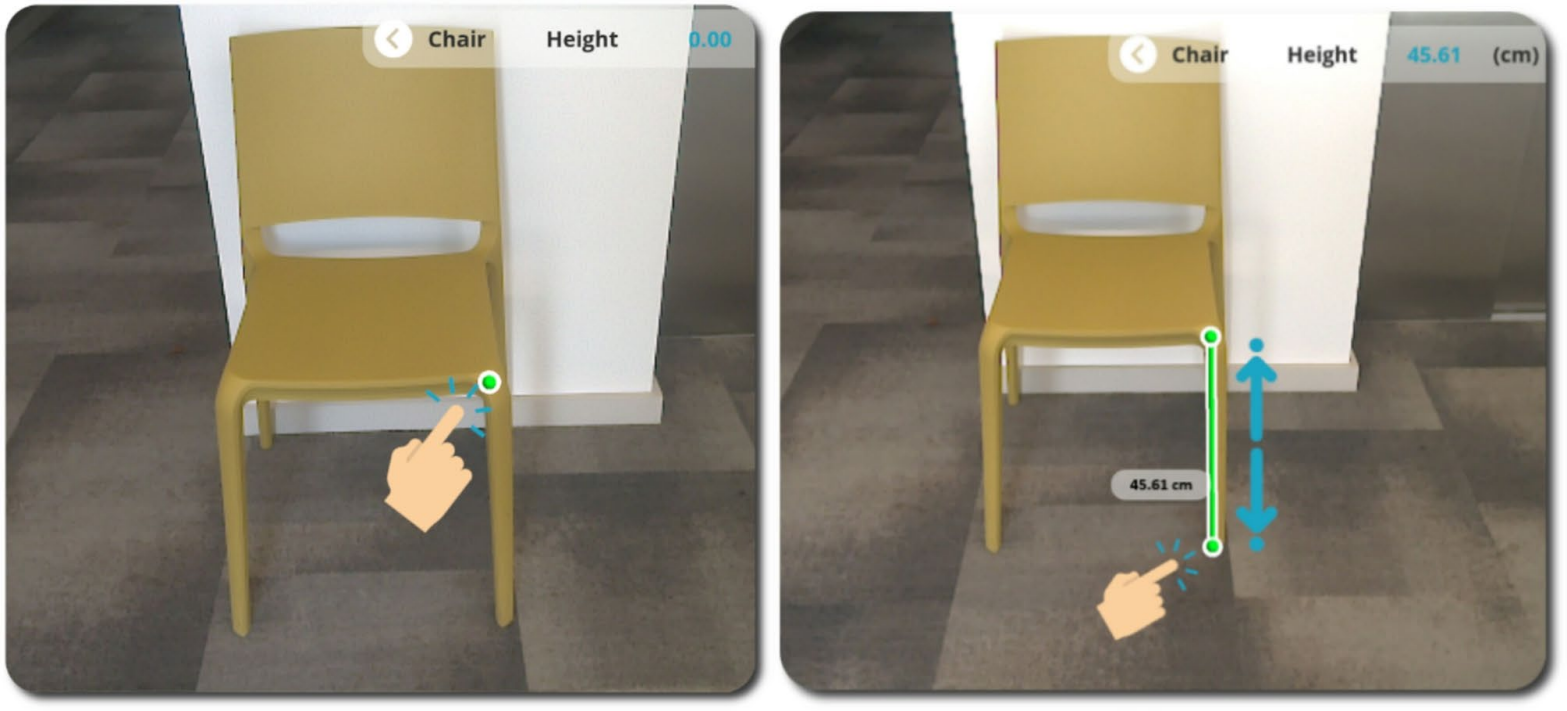
[Left: **a**] Marking a Measurement Point, [Right: **b**] 3D Line drawn in relation to the Time-of-Flight depth with the measurement result in an adjacent 3D Label

Users are able to measure objects that appear within the device POV by simply dropping a series of point-to-point measurement markers which are augmented into the scene. The mechanism for placing measurement markers is presented in Fig. 5.

**Fig. 6:**
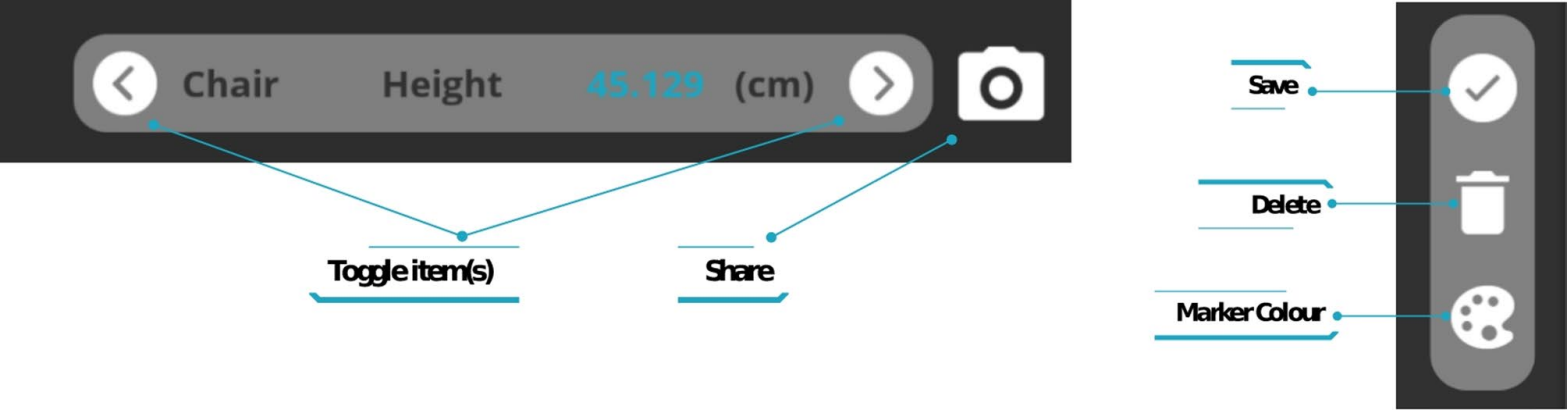
Application control panels, [Left] Measurement Indicator Overlay including buttons to toggle through furniture items, [Right] Measurement Controls Overlay to Save, Delete or adjust Marker Colour

The first step in taking on-screen measurements requires the user to point the device’s camera towards the item of interest in the physical world. The next step is to select the item name (i.e. bed, bath, chair, stairs, toilet, or popliteal height) and the measurement type (height, width, depth, etc.) that is to be measured. This is done by toggling through the Measurement Indicator Overlay control panel using the right and left arrow buttons until the required item name and measurement type is showing in the panel. Figure 6 presents a close-up view of the application control panels with the ‘Measurement Indicator Overlay’ panel on the left and ‘Measurement Controls Overlay’ panel on the right.

**Fig. 7:**
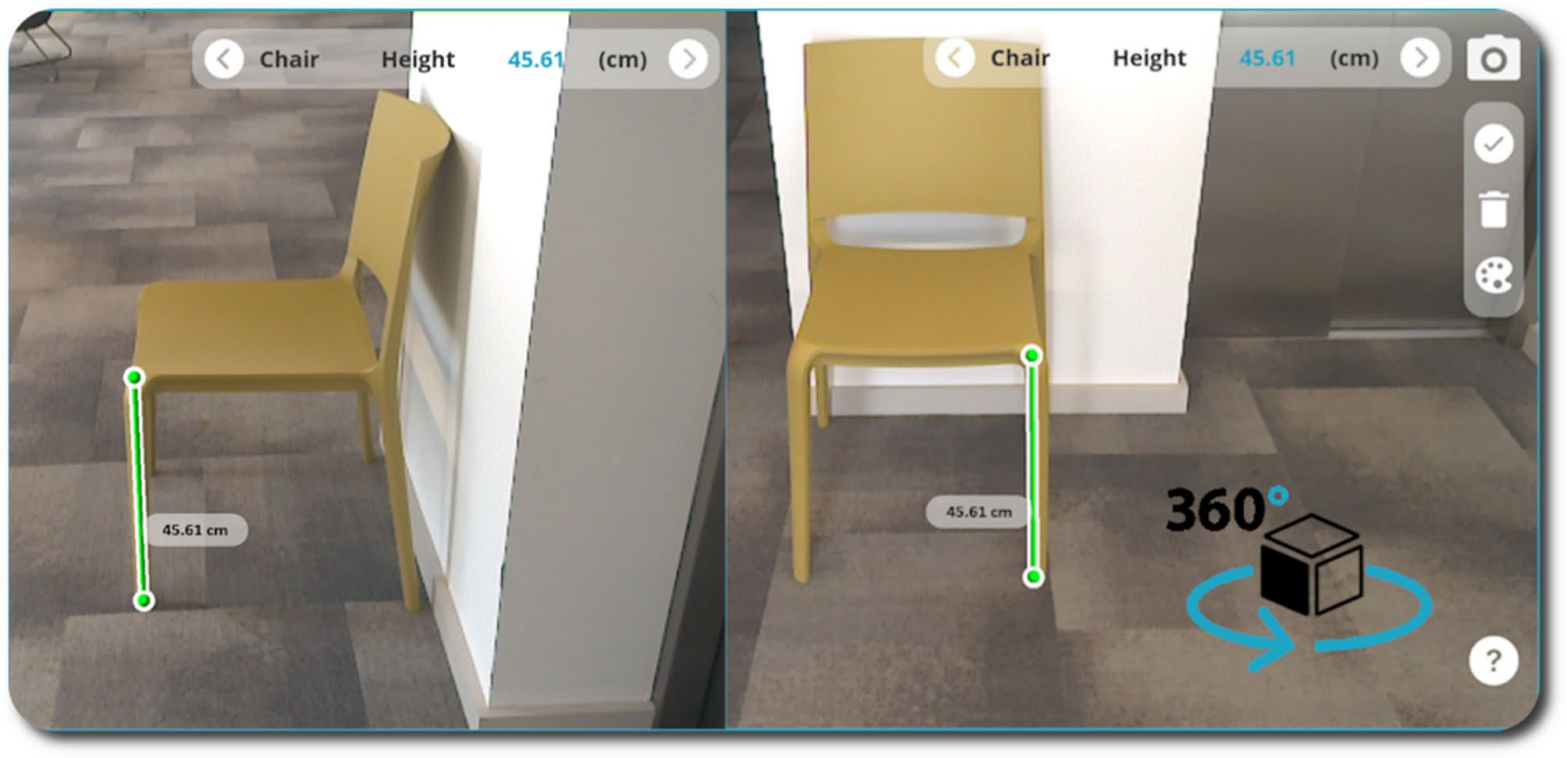
A Change in POV of the device whereby 3D Measurement markers are fixed in Euclidian World-Coordinate space in accordance with the Time-of-Flight depth results

The user is then able to drop an initial 3D marker onto the object within the camera view (shown as a green sphere in Fig. 5) by simply touching the object on-screen in the precise location that they would like the measurement to start from. The marker is immediately augmented onto the chosen object on-screen by mapping the Z-depth value obtained from the Time-of-Flight (ToF) sensor against the RGB camera intrinsic parameters (focal length and principal points) to obtain real world distance to 2D pixel coordinate mapping. The user can then place a second marker (i.e., the end point of the measurement) in a similar way the first marker was placed. Once both markers are placed, they are connected by a single straight line which indicates the precise point-to-point measurement that is being taken (shown as a green cylinder in Fig. 5). The single line is also accompanied by the measurement value in centimetres, which is calculated as the Euclidean distance between two 3D points and is presented in the form of a label positioned adjacent to the line. The measurement markers and the connecting line are anchored into the 3D map of the scene as interactable 3D objects, which have been placed relative to the device’s coordinate space. This means that even if the device POV changes, the markers maintain the position they were placed in within the scene and they remain in the same location relative to the width, height, and depth of the item of interest. This allows the user to rotate around the object they have placed measurement markers on, to verify and confirm that they have placed the markers in the intended position. Figure 7 shows how markers can be placed to measure chair height remain anchored in position regardless of the POV.

#### Saving, deleting and adjusting measurement markers

**Fig. 8:**
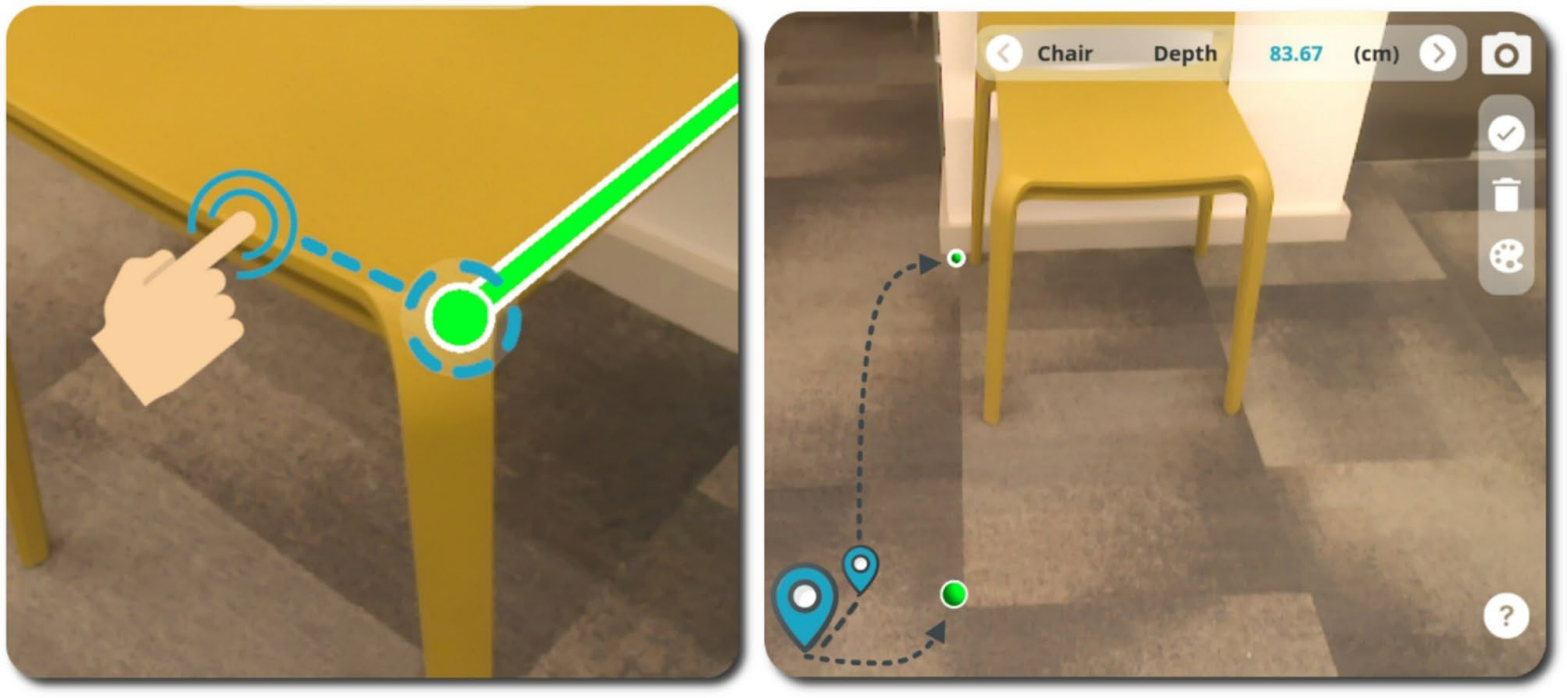
[Left] Adjusting a measurement marker by touching and dragging, [Right] Indication of depth through 3D object occlusion and size (note: the measurement connector has been disabled for illustration purposes)

The ‘delete’ and ‘accept’ buttons (as shown in Fig. 6) can be used to delete the placed markers (each press, deletes the most recently placed marker) or to accept the current measurement results and store these according to the item selected in the measurement guidance indicator. Alternatively, users have the option of synchronously adjusting the placement of the measurement-markers (by selecting/touching and dragging the marker to the optimum position) to optimise their position after inspecting the location of the markers from a range of viewpoints within the camera’s view. Figure 8 (left) shows how the user can make a synchronous adjustment to the placement of the measurement markers within PilOT-Measure by means of the touch and drag features. PilOT-Measure also provides the user with a sense of depth, distance, and marker placement within 3D space by using object occlusion and measurement marker size as depth cues. The smaller the marker size, the further away the marker has been placed in 3D space. This allows the user to get instant visual feedback cues on the location of each marker that they place within the scene. Figure 8 (right) shows how the sense of depth and distance is achieved via relative marker size.

### System architecture

**Fig. 9:**
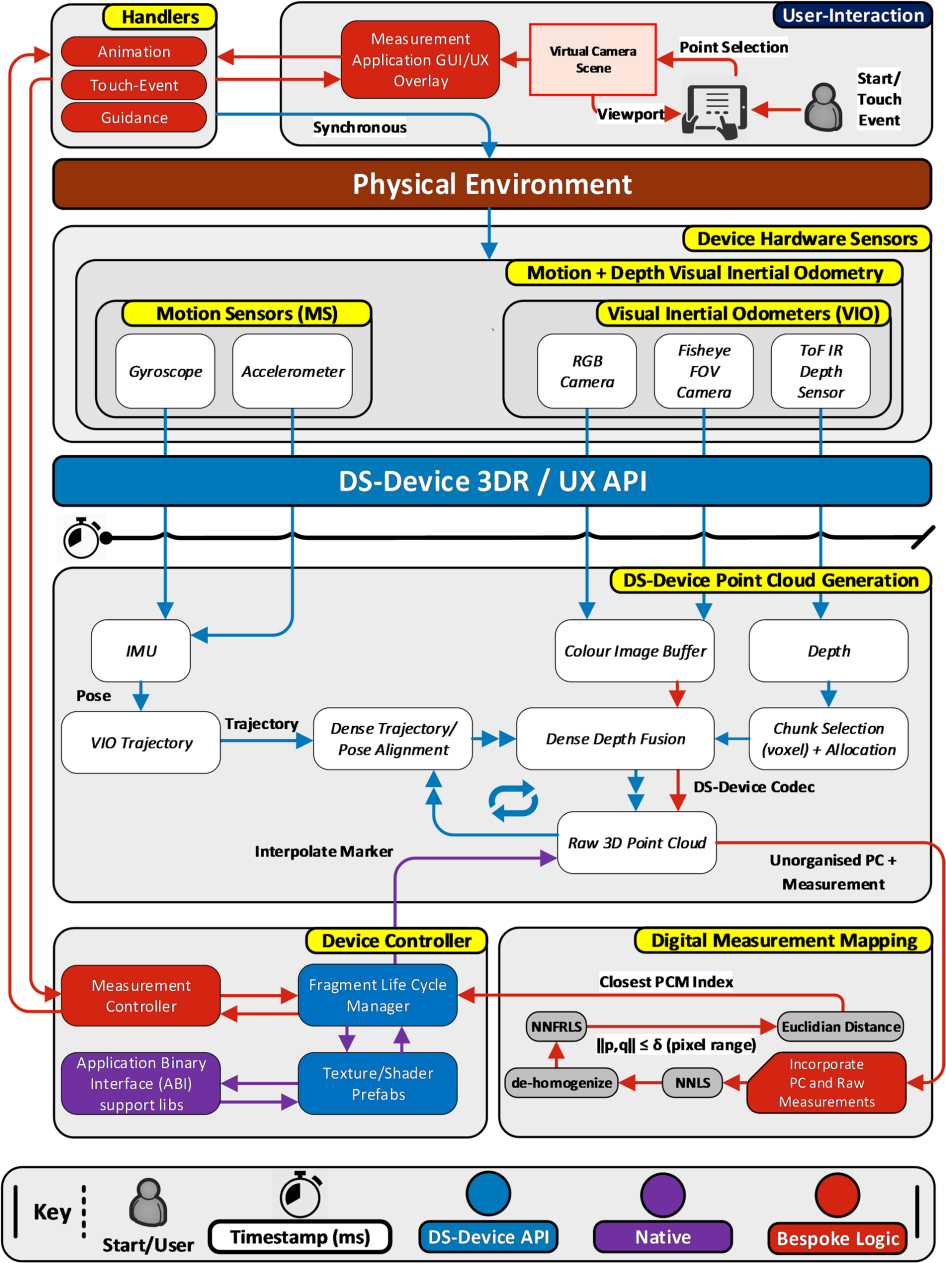
PilOT-Measure architecture diagram

This section provides a detailed overview of the PilOT-Measure system architecture. It also provides a formal presentation of the digital measurement mapping technique that has been developed specifically to enable the point-to-point measurement function on PilOT-Measure. Figure 9 presents an overview of the PilOT-Measure system architecture and digital measurement mapping technique.

In the first instance, the *Measurement Application GUI/UX Overlay* is used to initiate the process of scanning an environment (*Physical Environment*) and enabling the user to take point-to-point measurements of objects in that environment. A bespoke set of *Animation*, *Touch-Event* and *Guidance* objects are provided as user interface and data manipulation structures necessary for the user to carry out scans of the environment and record the required point-to-point measurements through a touch-enabled *Virtual Camera Scene* overlay. Recorded measurements are passed to the *Device Controller* that delegates low-level serialisation functions and assigns interpreters and pointers to handle managed objects from unmanaged memory space. The managed objects in this instance represent marshalled structures of the *Motion Sensor* (MS) and *Visual Inertial Odometers* (VIO) data objects. The *Device Controller* also handles the device’s lifecycle (i.e., how data is passed between objects and classes) and ensures buffer overflow exceptions are handled safely.

Concurrently, whilst the recorded measurements are delegated, the *Physical Environment* propagates the *Device Hardware Sensors* to scan the environment under inspection and capture associated raw data providing a formal digital representation of that environment. This typically includes data captured by the *Motion Sensor (MS)* unit (*Gyroscope* and *Accelerometer*), and *Visual Inertial Odometers* (VIO) (*RGB Camera*, *Fisheye FOV Camera* and *ToF-IR Depth Sensor*). Given that each respective *MS* and *VIO* sensor records at its own sampling rate, the *DS-Device 3DR API* and *DS-Device UX API* regulate the rate at which raw data is sampled and applies a system timestamp to keep track of data-points.

The *DS-Device Point Cloud Generation* component, which is typically provided as standard with the given device, processes the interpolated *MS* and *VIO* data via *IMU*, *Colour Image Buffer*, *Depth*, *VIO Trajectory*, *Dense Trajectory Pose Alignment*, *Dense Depth Fusion* and *Chunk Selection* to produce a *Point Cloud* (PC). Likewise, bespoke and feature dense open-sourced Application Programming Interfaces (APIs) exist that can generate and process PCs in similar fashion [40, 58, 59] whereby the algorithmic intrinsic is published and can be subject to further modification [55, 60, 61]. The processing carried out to produce the *PC* is in-line with the specifications of the *DS-Device Codec* that is deployed on the given device.

Upon completion, the *Point Selection* data, which is provided by the user as part of the point-to-point measurement task, is interpolated (*Interpolate Marker*) with the *PC* via the *Digital Measurement Mapping* that contains a tailored search algorithm and returns a corresponding index in the *PC* that represents the closest vertex. In this mapping, to avoid projective geometry anomalies (i.e. hovering, mismatched pixel and vertex points), we adopt standard mapping protocols for the ToF depth sensor ($\:{T}_{\varvec{a}}$) and RGB Colour Camera ($\:{C}_{\varvec{a}}$) data to obtain real world pixel-to-cm distance conversion. Specifically, the *PC* vectors $\:{{T}}_{{a}}\left({X},\:{Y},\:{Z},\:{W}\right)$ and it’s transformation matrix are extracted and mapped against the *Point Selection* data $\:{C}_{a}\left(\varvec{x}\right)$ and $\:{C}_{\varvec{a}}\left(y\right)$ by querying the focal length values $\:{C}_{fx}$ and $\:{C}_{fy}$ with reference to the principal points $\:{C}_{px}$ and $\:{C}_{py}$. The result of this work provides a 2D and 3D coordinate mapping that reflects the pixel and real-world vector coordinate systems. The final depth results are back-propagated through the marshalled structures and animated as interactable 3D UX elements.

Upon receiving the users mapped *Point Selection* at the *Digital Measurement Mapping* module, and in consideration of the *Interpolate Marker* function, a Nearest-Neighbour Fixed-Radius Linear Search (*NNFRLS*) algorithm is applied. The NNFRLS algorithm is presented in Table 1 including points of interest.

**Table 1:** NNFRLS algorithm

PSEUDO-CODE: NNFRLS 2D-3D Incorporation < Method>		
**INPUT: M <** PointCloudMatrix **> FORMAT** [X, Y,Z, W], **p** < x,y>, $\:\varvec{\delta\:}$ < int>		
**OUTPUT**: An integer index of the PCD closest to the user input vector		
**ACTIVATION**: User Touch-Event < single>, < drag>		
1	**SET** best_pcm_index = -1;	
2	**SET** best_sqr_ditance = 0;	
3		
4	**FOR** (v = 0 **TO** M.Count) **DO**	⊲(1)
5	**SET** screen_pos_3d = **Dehomogenise** (M[v]);	⊲(2)
6	**SET** screen_pos_2d = vector < screen_pos_3d.x, screen_pos_3d.y>;	
7	**SET** sqr_distance = SquareMag (screen_pos_3d - **p**)	⊲(3)
8	**IF** (sqr_distance >$\:\varvec{\delta\:}\varvec{*}\varvec{\delta\:}$) **THEN**	⊲(4)
9	CONTINUE;	
10	END IF;	
11		
12	**IF (**best_pcm_index == -1 || sqr_distance < best_sqr_distance**) THEN**	⊲(5)
13	**SET** best_pcm_index = v;	
14	**SET** best_sqr_distance = sqr_distance;	
15	END IF;	
16	END FOR;	
17	**RETURN** best_pcm_index;	

In Table 1, input is delivered to the NNFRLS algorithm whereby $\:M$ is an unorganised point-cloud data set in homogenous coordinate format [62], $\:p$ is the *Point Selection* marker in standard Cartesian coordinate format and $\:\delta\:\:\left(delta\right)$ represents a number of pixels for fixed-search considerations in integer format.

The NNFRLS algorithm presented in Table 1 therefore has five points of interest (⊲). At Point (1) we locally iterate through each point cloud vector, which commonly is referred to as a naïve (linear) search-based function. Subsequently at Point (2), the 4D Homogeneous coordinates, which are projections of geometric objects in a 3D space (i.e., unorganised point cloud vectors), are de-homogenized to provide spatial mapping in the local coordinate system for viewing and processing purposes. Homogenization is a common algebraic function to make the degree of every term the same and is an inexpensive transformation that is ubiquitously available across graphical platforms such as OpenGL, OpenAI, Unity, Maya, AutoCad, Unreal. Furthermore, at Point (3) the square magnitude of the resulting homogenised vector is computed against the input vector $\:p\left(x,y\right)$ and its result at Point (4) is subjected to a pixel distance $\:\delta\:$ such that $\:\left\|x,y\right\|\le\:\delta\:$ (whereby we find all pairs $\:\left(x,y\right)\in\:M$ by which the distance between $\:x$ and $\:y$ is no more than $\:\delta\:$). The result of Point (4) is used as an indication on whether to skip processing the current vectors and omit storing its index. Finally, at Point (5), a check is performed to verify whether the current vector is within the acceptable range and is smaller than our previously stored distance. Upon completion, an index $\:s$ of the $\:M$ set is returned that is closest to the input vector or a -1 if none were found that satisfy $\:\left\|x,y\right\|\le\:\delta\:$.

The NNFRLS algorithm is inspired by Dickerson and Drysdale (1990) [63] whom presented a pruning method that constructs the Delaunay triangulation for a given set of points. Considering the unorganised structure of $\:M$ [62], whereby we only require the adjoining vertex of the user’s point of interest (measurement) relative to the device’s (camera) projection matrix, constructing a Delaunay triangulation to examine every point such that no points circumcircle is inside the circumcircle of any triangle in the set, would be computationally inefficient since we only require a single point query. Consequently, given $\:v$ is a set of vector points in a space $\:M$ and query point $\:p\in\:M$ (*Point Selection*) we can distil the search-space by finding the closest point in $\:M$ to $\:p$. Typically, $\:M$ is in metric space and therefore dissimilarity is expressed as a distance metric that is symmetric and can satisfy triangle inequality. Particularly, $\:M$ in this instance is a d-dimensional vector space where dissimilarity can be measured through Euclidian distance or Manhattan distance. In accordance, the Nearest-Neighbour Linear proximity search (NNLS) for a given 2D vector relative to the de-homogenised vertices is conducted as described above. In addition to NNLS, and in the interest of marginal efficiency, a Fixed-Radius search is also applied whereby the NNLS search is limited to an adjustable search range that is based on the average size of the pointer finger set to 16–20 mm (45–57 pixels) [64].

## Methods

**Fig. 10:**
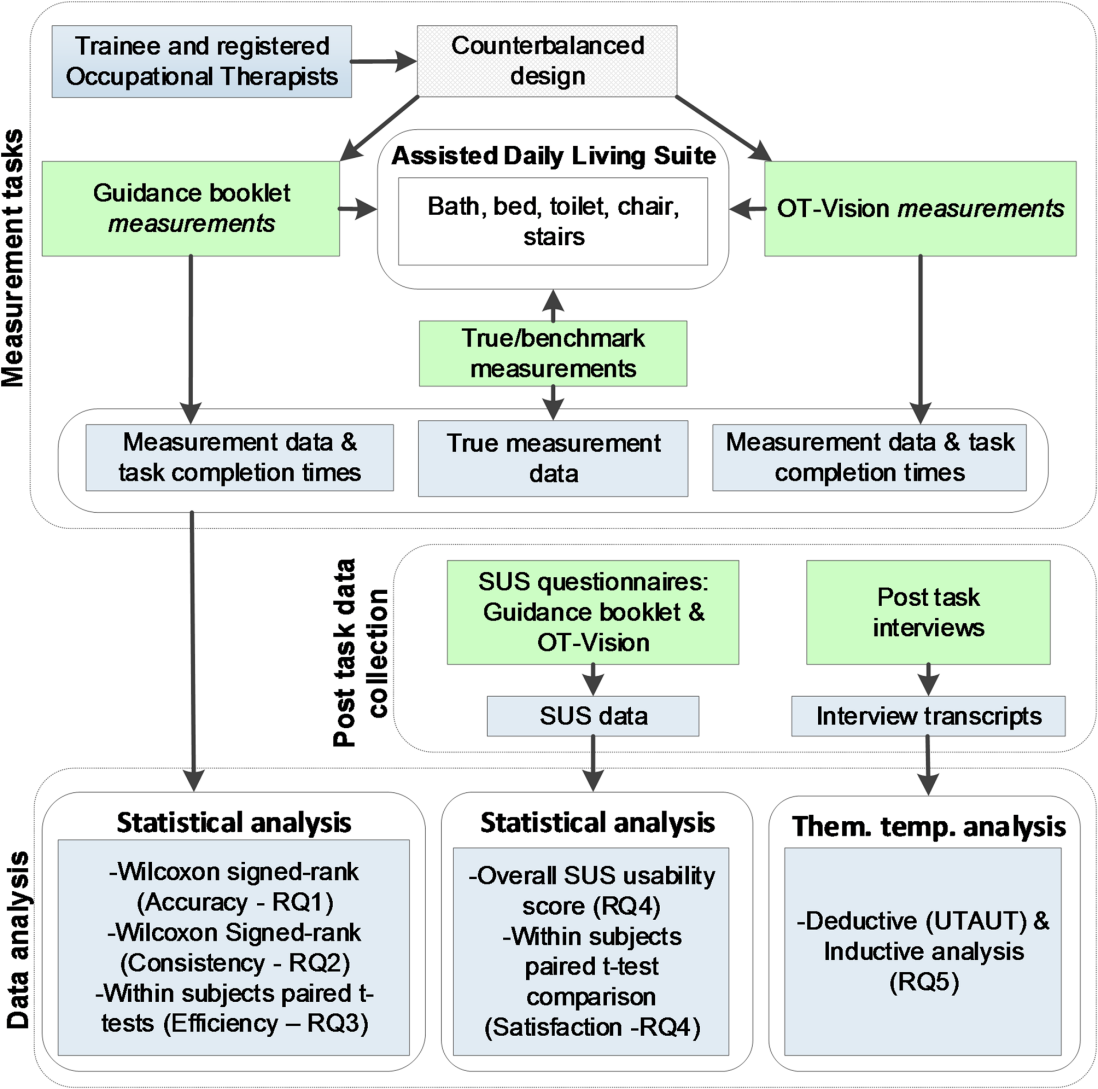
PilOT-Measure data collection and analysis protocol

This section provides details of the data collection and analysis protocol used to address the specific research aims of this study. Figure 10 provides an overview of the data collection and analysis protocol.

### Study participants

Twenty-one trainee and registered Occupational Therapists (OT) took part in this study. Initial recruitment involved approaching local NHS trusts and academic OT training facilities to invite staff to take part. Additional invitations were distributed on OT social network pages such as Facebook, LinkedIn and Academic Intranets that engage with home adaptations specialists. Subsequently, a chain-referral sampling approach was adopted which involved existing participants disseminating the invitation to colleagues. The inclusion criteria were that participants: (1) are familiar with the usage of smartphone enabled technologies such as tablets, and mobile phones; (2) are considered to be active with no restrictions on their ability to follow instructions related to key furniture measurements as identified by the measurement guidance booklet: (3) have experience in the provision of assistive equipment and minor adaptions, or have carried out home visit assessments; (4) were proficient English speakers. The demographic details of the participants reveal that the majority were female (85.7%, *n* = 18). Although there is a significant gender imbalance in this sample, its appropriateness may be, to some extent, justified by the view that occupational therapy tends to be very much a female-dominated profession [65, 66]. A summary of participant demographics details are presented in Table 2.

**Table 2:** Participants demographics

ID	Role	Age	Gender	Specialism/Work/Experience	Career Level
PP-1	Participant	34	F	Associate OT Researcher	5 + years
PP-2	Participant	25	F	NHS Community OT Specialist Trainee	2 years
PP-3	Participant	37	F	NHS Community Staff, Senior Research Staff	10 + years
PP-4	Participant	26	M	American Society of Physical Therapy Clinician	5 + years
PP-5	Participant	22	M	NHS 1st Round Community OT Trainee	1 year
PP-6	Participant	30	F	NHS 1st Round Community OT Trainee	1 year
PP-7	Participant	29	F	NHS 1st Round Community OT Trainee	3 years
PP-8	Participant	35	F	NHS 1st Round Community OT Trainee	1 year
PP-9	Participant	36	M	NHS 1st Round Community OT Trainee	1 year
PP-10	Participant	31	F	NHS 1st Round Community OT Trainee	5 + years
PP-11	Participant	41	F	NHS 1st Round Community OT Trainee	5 + years
PP-12	Participant	28	F	NHS 1st Round Community OT Trainee	1 year
PP-13	Participant	28	F	NHS 1st Round Community OT Trainee	1 year
PP-14	Participant	27	F	NHS 1st Round Community OT Trainee	1 year
PP-15	Participant	33	F	NHS 1st Round Community OT Trainee	1 year
PP-16	Participant	20	F	NHS 1st Round Community OT Trainee	1 year
PP-17	Participant	39	F	NHS 1st Round Community OT Trainee	1 year
PP-18	Participant	24	F	NHS 1st Round Community OT Trainee	1 year
PP-19	Participant	NA	F	NHS 2nd Round Community OT Trainee	5 years
PP-20	Participant	NA	F	NHS 3rd Round Community OT Trainee	5 + years
PP-21	Participant	23	F	NHS 1st Round Community OT Trainee	3 years

### Protocol and instrumentation

A mixed-methods counterbalanced within-subjects experimental design was adopted to verify the accuracy and consistency of the measurements recorded using PilOT-Measure and the manual tape measure. The study was conducted in a controlled Assisted Daily Living (ADL) suite at Brunel University London and St’ Georges University London. The ADL suite hosted a bathroom (with toilet and bath), bedroom (with bed and chair), and full-length stairs. In preparation for the trials, the ADL suite was assembled by expert technicians to represent a typical daily living environment whilst ensuring that all necessary items were in place for the measurement task. To serve as a benchmark for verification and validity purposes, the ‘True’ measurements were taken for each item that would later be measured in the trials. To establish true measurements, four expert clinicians took measurements for each item and reached consensus on the true mean values against which measurements recorded by participants could be compared. Informed consent was obtained prior to the study and at the start of each session. During the study, participants were given a brief demonstration of the two measurement tools (i.e. PilOT-Measure, and booklet with tape measure) and were given a tour of the ADL suite if they were not already familiar with the layout. They were then issued with one of the measurement tools, i.e. either PilOT-Measure or a tape measure and asked to record the measurements of items as indicated by the measurement guidance in the booklet. For both tools, participants were able to locate the start and end points for each measurement taken by using to the measurement guidance provided in the booklet. When using PilOT-Measure, participants had access to a digital version of the measurement guidance provided in the booklet. This provided them with guidance on how and where to locate measurement points for each respective item regardless of whether they were using the tape measure or PilOT-Measure to take measurements. During this process the total amount time taken to carry out each measurement task was recorded. Once the measurements were taken, participants were asked to complete a System Usability Scale (SUS) questionnaire [67] which included 10 standard questions about the their experience of using each of the respective measurement tools [68]. Participants were required to rate all SUS statements using a 5-point Likert type scale ranging from 1 (strongly disagree) to 5 (strongly agree). All participants then performed a second iteration of this procedure, using the alternative measurement guidance tool. A counterbalanced design was used to control for the order effects. Upon completion of all tasks and SUS questionnaires, a semi-structured post-task interview was conducted with each participant. The interview consisted of a set of closed and open-ended questions (see supplementary file) to capture the user’s outlook on the perceived usefulness, challenges, and opportunities which were recorded and transcribed verbatim.

### Data analysis

The IBM SPSS statistics package Version 26.0 was used to analyse the measurement data, task completion times, and SUS questionnaire survey responses. Measurement error values were calculated as the difference between participant measurement values and corresponding true measurement values. One-sampled Wilcoxon signed-rank tests were applied to verify measurement accuracy (RQ1) i.e., whether the median error differences were significantly different from the true values for each measurement tool respectively. Error values were converted to absolute error values. To establish whether there was a significant difference between the two measurement tools, in terms of the accuracy consistency (RQ2), the related samples Wilcoxon signed-rank test was applied to compare the ranked differences of absolute error values generated by both tools. The Wilcoxon signed rank test was conducted as the datasets were not normally distributed. Paired sample t-tests were applied to test for differences in task completion times (R3) and to compare differences in individual SUS item responses (R4) and the two subscales that SUS is said to be made up of i.e. Usability (SUS items 1–3, 5–9) and Learnability (SUS items 4 & 10) [68]. Furthermore, overall SUS scores were calculated and interpreted according to the acceptability range, and the adjective and school grading scales [68]. This involved calculating a mean SUS representative value on a 100-point rating scale for each sample. These scores were then mapped to descriptive adjectives (Best imaginable, Excellent, Good, OK, Poor, Worst Imaginable), an acceptability range (Acceptable, Marginal-High, Marginal-Low, Not acceptable) and a school grading scale (i.e. 90–100 = A, 80–89 = B etc.). The baseline adjective and acceptability ranges are derived from a sample of over 3000 software applications [68].

The post-task interview data (RQ5) was perused using a Thematic Template Analysis approach [69] whereby specific extracts from the data are coded and analysed both inductively, whereby data drives the development of themes, and deductively, whereby a set of priori (pre-defined) themes are linked to analytical interests of researches through theory driven approaches [70, 71]. The first stage comprised of generating a template constructed on the three key factors of technology use and adoption defined by the Unified Theory of Acceptance and Use of Technology (UTAUT) Model [72]. The factors include: Performance Expectancy (PE); Effort Expectancy (EE); Social Influence (SI) and help to determine if (RQ5) an individual will adopt or reject a new system. The second stage perused the entire corpus and coded specific extracts from the data related to the three UTAUT themes by which other high-level themes emerged, and similar text groupings were formulated by moving, placing and re-reading segments to ensure groupings were warranted and substantiated. The third stage iteratively repeated the perusal of the corpus and spliced, linked, deleted and reassigned text to subsequent high-level themes and subthemes. The final template covering the themes in totality is congruent with ‘contextual constructivism’, a stance formulated on the premise that there are various interpretations of a given observable occurrence that is dependent on the context of the data capture, collection and analysis [73, 74].

## Results

### Measurement accuracy

The first research question was to compare the accuracy of the measurement results recorded by PilOT-Measure and in the paper-based booklet respectively. Measurement median error difference values were calculated as the difference between the booklet or digital PilOT-Measure measurement values, and the true values. The results of the comparison between the PilOT-Measure and the booklet, and the extent to which the respective recorded measurements are significantly different from the true measurement values, are presented in Table 3.

**Table 3:** Measurement accuracy for PilOT-Measure vs. Booklet

	True (cm)	PilOT-Measure				Booklet				
		Md (cm)	Md Diff. (cm)	Z	Sig. (2-tail)	Md (cm)	Md Diff. (cm)	Df	Z	Sig. (2-tail)
**Bath**										
Height	45.58	45.00	-0.58	1.373	0.170	45.07	-0.51	20	2.07	0.038*
Int W.	57.60	57.50	-0.10	2.485	0.013*	54.19	-3.41	20	1.50	0.134
Ext W.	69.67	70.00	0.33	-1.373	0.170	70.20	0.53	20	-1.77	0.076
Length	166.57	166.70	0.13	-1.964	0.050*	168.10	1.53	20	-0.16	0.875
**Bed**										
Height	53.65	53.00	-0.65	-2.207	0.027*	56.47	2.82	20	0.57	0.566
**Chair**										
Height	45.60	48.00	2.40	-1.755	0.079	46.90	1.30	20	-2.96	0.003*
Depth	44.50	44.00	-0.50	1.547	0.122	43.43	-1.07	20	0.84	0.400
Width	42.35	41.91	-0.44	-1.269	0.205	42.41	0.06	20	0.30	0.767
**Toilet**										
Height: A	48.75	48.00	-0.75	0.191	0.848	49.40	0.65	20	3.14	0.002*
Height: B	46.40	45.50	-0.90	-0.226	0.821	46.42	0.02	20	2.68	0.007*
**Stairs**										
Length	85.00	85.00	0.00	-1.912	0.056	85.89	0.89	20	0.24	0.812

* Indicates statistically significant at < = 0.05 level

#### Measurement accuracy - results summary

According Table 3, which presents the measurement accuracy results, the median differences (denoted *Md Diff.*) between the two measurement guidance tools, in 6 out of the 11 cases, PilOT-Measure delivered the smallest median difference, compared with the booklet. Therefore, as an initial observation, this suggests that, in absolute terms, PilOT-Measure tended to generate more precise (but not necessarily accurate) measurements compared to those recorded in the booklet.

The one sampled comparison of PilOT-Measure’s observed median values against the true measurement, reveals that eight out of 11 cases of the median error differences are not significantly different from the true measure: Bath Height (*z* = 1.373, *p* = 0.17), Bath External Width (*z* = -1.373, *p* = 0.17), Chair Height (*z* = -1.755, *p* = 0.079), Chair Depth (*z* = 1.547, *p* = 0.122), Chair Width (*z* = -1.269, *p* = 0.205), Toilet Height A (Floor-bowl) (*z* = 0.191, *p* = 0.848), Toilet Height B (Floor-seat) (*z* = -0.226, *p* = 0.821), Stairs Length (*z* = -1.912, *p* = 0.056). This indicates that in these cases, there is no evidence that PilOT-Measure produces inaccurate measurements at the < = 0.05 significance level. Three cases out of 11 were significantly different from the true measure, suggesting that in these cases, PilOT-Measure produced inaccurate measurements at the < = 0.05 significance level.

The one sampled comparison of the booklets’ observed median values against the true measurement, reveals that seven out of 11 cases of the median error differences are not significantly different from the true measure: Bath Internal Width (*z* = 1.497, *p* = 0.134), Bath External Width (*z* = -1.772, *p* = 0.076), Bath Length (*z* = -0.157, *p* = 0.875), Bed Height (*z* = 0.574, *p* = 0.566), Chair Depth (*z* = 0.841, *p* = 0.4), Chair Width (*z* = 0.296, *p* = 0.767), Stairs Length (*z* = 0.238, *p* = 0.812). Four of the 11 cases were significantly different from the true measure, indicating that in these cases, the booklet produced inaccurate measurements at the < = 0.05 significance level.

Overall, comparing the performance of the two conditions, PilOT-Measure produced inaccurate measurements for three out 11 items whereas the booklet produced four out of 11 items. The items in both conditions differ, with the booklet producing one more inaccurate result. Furthermore, for cases where PilOT-Measure and the booklet provided accurate measurement with no statistically significant difference: Bath External Width, Chair Depth and Stair Length measurements, PilOT-Measure delivered smaller median differences for all items.

In terms of items, PilOT-Measure has produced statistically accurate values for all Bed, Chair, Toilet and Stairs measurements, however failed to do so with similar effect in the Bath. The booklet has generated three out of the four bath measurements accurately (Internal Width, External Width and Length), whereas PilOT-Measure did so for two out of the four (Height and External Width). Despite this, in absolute terms the median error difference for the PilOT-Measure was smaller compared with the booklet for the Bath specifically with exception of the Bath height.

In addition, the booklet provided statistically inaccurate results for all Toilet cases when compared to the true measure: Toilet Height A (*p* = 0. 002), Toilet Height B (*p* = 0.007) which was not the case for PilOT-Measure, which produced measurements that were not significantly different from the true median. To this end, the biggest median measurement differences were identified in the booklet: Bath Internal Width (-3.41 cm), Bath Length (1.53 cm) and Bed Height (1.30 cm), of which the Chair height statistically different from the true measurement at the < = 0.05 significance level.

### Measurement accuracy consistency

The second research question was to compare the accuracy consistency of measurements recorded using the two respective guidance tools. The results of the PilOT-Measure and Booklet analysis are presented in Table 4.

**Table 4:** Measurement accuracy consistency for PilOT-Measure app vs. Booklet

	PilOT-Measure	Booklet	Paired Differences					
	Abs.Md.err (cm)	Abs.Md.err (cm)	Md.err.diff (cm)	Df	Z	Sig. (2-tail)	Effect size (*r*)	Effect size mag.
**Bath**								
Height	1.23	0.58	0.65	20	-1.390^a^	0.164	0.311	Medium
Int W.	4.83	0.60	4.23	20	-3.632^a^	0.000*	0.812	Large
Ext W.	1.85	0.33	1.52	20	-2.242^a^	0.025*	0.501	Large
Length	2.43	0.43	2.00	20	-2.694^a^	0.007*	0.602	Large
**Bed**								
Height	3.50	2.15	1.35	20	-2.520^a^	0.012*	0.563	Large
**Chair**								
Height	1.96	2.40	-0.44	20	− .226^a^	0.821	0.051	Trivial
Depth	3.44	3.50	-0.06	20	− .859^a^	0.391	0.192	Small
Width	1.69	1.85	-0.16	20	− .556^a^	0.578	0.124	Small
**Toilet**								
Height A	1.92	0.75	1.17	20	-2.398^a^	0.016*	0.536	Large
Height B	1.31	0.90	0.41	20	-2.207^a^	0.027*	0.494	Medium
**Stairs**								
Length	1.21	0.95	0.26	20	-1.547^a^	0.122	0.346	Medium

a Based on negative ranks

* Statistically significant at < = 0.05 level

#### Measurement accuracy consistency - results summary

According to the results presented in Table 4, in two of the 11 cases, the median error value for the booklet was larger than the PilOT-Measure equivalent, hence resulting in a negative median error difference (denoted *Md.err.diff*) between PilOT-Measure and booklet: Chair Height (*Md err. diff*. = -0.44), Chair Width (*Md err. diff* = -0.16). In the remaining nine cases, the median error for the booklet was smaller than PilOT-Measure app, resulting in a positive median error difference: Bath Height (*Md.err.diff* = 0.65), Bath Internal Width (*Md.err.diff* = 4.23), Bath External Width (*Md.err.diff* = 1.52), Bath Length (*Md.err.diff* = 2.00), Bed Height (*Md.err.diff* = 1.35), Chair Depth (*Md.err.diff* = -0.06), Toilet Height A (*Md.err.diff* = 1.17), Toilet Height B (*Md.err.diff* = 0.41) and Stairs Height (*Md.err.diff* = 0.26). This indicates that the mid-point error values tended to be lower for the booklet when compared with PilOT-Measure.

The Wilcoxon signed-rank test comparing the absolute error differences of PilOT-Measure app and the booklet measurements, reveals that in six out of the 11 cases that are statistically significant, PilOT-Measure app less consistently produced accurate measurements than the booklet: Bath Internal Width (*z* = -3.632b, *p* = 0 with Large-effect size), Bath External Width (*z* = -2.242b, *p* = 0.025 with Large-effect size), Bath Length (*z* = -2.694b, *p* = 0.007 with Large-effect size), Bed Height (*z* = -2.520b, *p* = 0.012 with Large-effect size), Toilet Height A: Floor-bowl (*z* = -2.398b, *p* = 0.016 with Large-effect size), Toilet Height B: Floor-seat (*z* = -2.207b, *p* = 0.027 with Medium-effect size).

All *z* scores were based on negative ranks, which further confirms that which was indicated by the negative median error differences, that in the majority of cases (nine of the 11) the sum of ranked negative differences was lower than the sum of positive ranked differences indicating that booklet consistently produced more accurate measurements (i.e. lower measurement error differences) compared with PilOT-Measure.

Overall, comparing the performance of PilOT-Measure and booklet in terms of accuracy consistency, the booklet outperformed PilOT-Measure in six of the 11 cases. In the remaining five cases, although the differences were not significantly different in statistical terms, three cases (Chair Height, Depth, Width) resulted in the booklet generating a larger error difference and the remaining two (Bath Height and Stair Length) generating error differences all under one centimetre. The smallest observed difference was for the Chair Depth, which generate a difference of 0.06 cm between the booklet and PilOT-Measure app. Although not significant, it is also interesting to observe the Chair to be the only consistently accurate measurement.

### Task completion time

The third research question was to consider whether there are any significant differences in the task completion time (measured in seconds) for each measurement item when using the respective measurement guidance tools. The results of analysis are presented in Table 5.

**Table 5:** Task completion time for PilOT-Measure app vs. Booklet

	PilOT-Measure Mean (Sec.)	Booklet Mean (Sec.)	Mean Diff. (Sec.)	St. Dev	t	Df	Sig (2-tail)
**Bath**							
Height	12.39	10.26	-2.13	6.681	-1.461	20	0.160
Int W.	9.36	43.58	34.22	9.855	15.912	20	0.000*
Ext W.	11.04	8.46	-2.58	4.498	-2.629	20	0.016*
Length	6.90	21.81	14.91	5.915	11.550	20	0.000*
**Bed**							
Height	6.47	15.46	8.99	8.797	4.682	20	0.000*
**Chair**							
Height	11.10	14.99	3.90	6.492	2.750	20	0.012*
Depth	12.16	14.67	2.51	7.054	1.628	20	0.119
Width	9.99	13.64	3.65	5.745	2.914	20	0.009*
**Toilet**							
Height A	14.71	14.72	0.02	5.634	0.012	20	0.990
Height B	29.15	17.16	-12.00	14.754	-3.727	20	0.001*
**Stairs**							
Length	11.15	28.21	17.07	7.087	11.035	20	0.000*

* Indicates statistically significant at < 0.05

#### Task completion time: results summary

According to Table 5 which presents the results of the paired samples t-test comparing the task completion times for PilOT-Measure and the booklet guidance, eight out of 11 cases were significantly different. In six out of 11 cases, participants required significantly more time to complete the task when using the booklet: Bath Internal Width (*M* = 43.58, *SD* = 9.86, *p* = 0.000), Bath Length (*M* = 21.81, *SD* = 5.92, *p* = 0.000), Bed Height (*M* = 15.46, *SD* = 8.8, *p* = 0.000), Chair Height (*M* = 14.99, *SD* = 6.49, *p* = 0.012), Chair Width (*M* = 13.64, *SD* = 5.74, *p* = 0.009), Stairs Length (M = 28.21, SD = 7.09, *p* = 0.000). The remaining two cases, resulted in the mean difference for PilOT-Measure being larger than that for the booklet, hence resulting in negative mean differences: Bath External Width (M = 8.46, SD = 4.45, *p* = 0.016) and Toilet Height B: Floor-seat (M = 17.16, SD = 14.75, *p* = 0.001).

In the three out of 11 cases that are not statistically significant, two resulted in the booklet requiring more time to complete the measurement tasks when compared to PilOT-Measure: Chair Depth (M = 14.67, SD = 7.05, *p* = 0.119) and Toilet Height A: Floor-bowl (M = 14.72, SD = 5.63, *p* = 0.99).

One additional observation that was made involved the measurement items considered to be the most cumbersome in terms of the clinician’s physical effort and item measurement distance, was that both the Bath and Stairs length resulted in statistically significant positive mean differences further indicating that PilOT-Measure overall produced faster results in the majority of the measurement tasks.

Overall, it is clear to assess the time completion performance to be in favour of PilOT-Measure in 6 out of 11 cases where the remaining non-significant cases still performed in favour of PilOT-Measure in 2 instances.

### Satisfaction and overall usability

The third research question was to evaluate the usability of the entire application compared with the booklet. The overall SUS score for application was 76.0 out of 100, which, according to the evaluation criteria for SUS [68], indicates that the application delivers ‘Good’ (Descriptive adjective), ‘acceptable’ (Acceptability range), and ‘Grade B+’ (School grading scale) levels of usability. The overall SUS score for the booklet was 58.5 out of 100, indicating ‘OK, ‘low marginal, and ‘Grade F’ levels of usability.

Follow-up analysis of individual SUS items for the application and the booklet were conducted to identify any specific usability issues that the participants experienced during the interactive task. Table 6 presents the individual SUS item results, differences (denoted as gap score) and corresponding significance values.

**Table 6:** PilOT-Measure app vs. Booklet SUS score comparison

SUS Items	PilOT-Measure Mean	Booklet Mean	Gap Score	Df	t	Sig. (2-tail)
S1: I think that I would like to use the app/booklet frequently.	3.86	2.95	0.90	20	2.528	0.020*
S2: I found the app/booklet unnecessarily complex.^a^	4.62	3.43	1.19	20	7.278	0.000*
S3: I thought the app/booklet was easy to use.	3.90	3.43	0.48	20	2.500	0.021*
S4: I think that I would need the support of a technical person to be able to use the app/booklet.^a^	4.48	3.81	0.67	20	3.005	0.007*
S5: I found the various functions in the app/booklet were well integrated.	3.67	3.24	0.43	20	1.686	0.107
S6: I thought there was too much inconsistency in the app/booklet.^a^	3.76	3.29	0.48	20	1.520	0.144
S7: I would imagine that most people would learn to use the app/booklet very quickly.	3.95	3.33	0.62	20	1.813	0.085
S8: I found the app/booklet very awkward to use.^a^	4.05	2.43	1.62	20	4.117	0.001*
S9: I felt very confident using the app/booklet.	3.67	3.48	0.19	20	0.847	0.407
S10: I needed to learn a lot of things before I could get going with the app/booklet.^a^	4.43	4.00	0.43	20	1.672	0.110*

^a^ Responses of negative items reversed to align with positive items, higher scores indicate positive responses

* Indicates statistically significant at < 0.05 level

#### Satisfaction and overall usability: results summary

According to the results resented in Table 6, all 10 SUS individual mean item scores were above the neutral mid-point of 3.00 for both the booklet and PilOT-Measure, indicating that overall, participants tended to be positive about PilOT-Measure and booklet for all items. In all cases, PilOT-Measure achieved higher absolute mean scores compared with the booklet, which is signified by the positive gap scores. This further indicates that for all of the ten SUS items, participants tended to be more positive about the application compared with the booklet. Whilst the participants tended to respond more positively for the application compared with the booklet in relation to SUS items S5, S6, S7, and S9, the differences however in statistical terms were not significant. Six of the ten SUS items: S1-S4, S8 and S10 were significantly different, and in all these cases, the application significantly outperformed the booklet. Above all, participants tended to be more enthusiastic about the application and felt that it delivered an improved user experience in in relation to conducting their practical work with attention of the usability and learnability constructs. Notwithstanding, the general trend inferred through the descriptive statistical results, an observed positive trend in the applications digital capabilities as a proxy for field work was substantial.

Results for item S1, reveal that participants tended to be more positive about the application and would prefer to use PilOT-Measure more frequently (*p* = 0.020). Item S2 further indicated that participants felt that PilOT-Measure was significantly less unnecessarily complex than the tape measure and booklet (*p* = 0.000). Responses for S3, show that participants found the application to be significantly easier to use compared to the booklet (*p* = 0.021). For S4, participants responded that using the application is significantly less likely to require the support of a technical person to be able to use it compared to using the booklet (*p* = 0.007). Results for item S8 suggest that participants agreed with finding PilOT-Measure was less awkward to use compared with the booklet (*p* = 0.001) and item S10 further suggest that participants did not feel like they needed to learn a lot before using PilOT-Measure (*p* = 0.110).

### Perceived challenges, opportunities, adoption and use

Six high-level themes emerged from the thematic analysis. Three of these themes emerged from deductive thematic template analysis related to the UTAUT model: Performance Expectancy; Effort Expectancy; Social Influence. The remaining three high-level themes emerged from inductive thematic analysis: Augmenting Equipment Provision; Clinical Self-Assessment; Privacy. The unique Participant ID, gender and age is included in parentheses alongside quotes from the analysed interview transcripts. A summary of the results is presented in Table 7.

**Table 7:** Summarised outcomes of thematic analysis

Theme	PilOT-Measure summarised outcomes
Performance Expectancy	More efficient for record keeping and note taking
	More accurate measurement in line with guidelines, delivering reporting efficiencies
	Facilitates inter-professional and joint decision making
	Remote measurement delivers health and safety benefits to patient and practitioner
Effort Expectancy	Intuitive user interface
	Challenges with measurement marker placement
	Improve marker placement support by augmented line edges on-screen
Social Influence	Potential to use PilOT-Measure for automated assessment or patient self-assessment
	Patient/practitioner age and experience possible barriers to adoption
Augment Equipment Provision	Potential to use digital images as visual aid for joint decision making
	Enhance functionality by visualising adaptations
Clinical Self-Assessment	Good potential as tool for patient self-assessment
	Concerns about accuracy of measurements
Privacy	Privacy concerns about the recording of digital images within the patient’s home

#### Performance expectancy

Participants reported that PilOT-Measure could serve as a valuable tool for the measurement guidance, pre-assessment and the initial assessment tasks that OTs engage with as part of the FRA. The fact that PilOT-Measure keeps a digital record of the home environment and the measurements taken was perceived as having the potential to reduce the stress of having to keep records and notes of home visits manually.

*…I think that will really help with ergonomic workload and it will help reduce stress … and there’s just so many things we need to measure quantitively and qualitatively as an OT … so for initial interviews and initial assessment this will be a very great tool. (PP7*,* 29*,* Female)*

The administrative overhead of taking down precise measurements of items around the patient’s home was noted as being particularly time consuming. One participant suggested that, often to reduce the overhead of taking precise measurements, clinicians round measurement values up or down (for example to the nearest five of 10 cm), which in turn affects the accuracy of the measurements taken. PilOT-Measure was seen as offering a valuable alternative that could help save time and maintain the accuracy of measurements by removing the temptation/need to round measurement values.

*I think it is a lot more precise*,* … you don’t want to do the mental maths to figure out the spots in between so you kind of just round it up… It’s nicer this way*,* it’s a nice precise answer. (PP3*,* 37*,* Female)*

Also, there was some perceived efficiency seen in taking depth-enabled measurements on-screen and recording these in digital format. These digital values could then potentially be easily integrated into an automatically generated report which in turn would remove the overhead of having to write down measurement values during the visit and then collate them into a written report to send to relevant stakeholders later.

*I do envision it as becoming a crucial tool. Lots of OT’s struggle with the basic maths measurements and do not perform them according to our guidelines [e.g. rounding up or down] … if we can have the measurement calculated*,* stored and sent off automatically [in a report] then that will make our lives a lot easier (PP1*,* 34*,* Female)*.

Participants also reported that the digital images of the home environment, that PilOT-Measure keeps a record of when taking measurements, has great potential to support inter-professional collaboration and joint decision making once the FRA visit has been completed, rather than attempting to collaborate with colleagues based on a verbal/written descriptive of the home environment.

*We always work as part of a team*,* so I think regardless of whatever equipment we get*,* there always is that element of maybe I should still confer with the team to get a 2nd opinion*,* especially for someone who starts at a Band 5. I see even Band 7 or 8’s they still come back and talk to the rest of the team. (PP3*,* 37*,* Female)*

It was suggested that PilOT-Measure’s ability to take measurements without having to make any physical contact with the items being measured, delivered several health and safety benefits. Some noted benefits included not having to kneel on the floor; not having to touch potentially unhygienic surfaces in the bathroom; and avoiding potential injuries from using measuring equipment.

*…using an application like this you don’t need to kneel-down…and in terms of hygiene… somebody might have just used the toilet…. you don’t need to touch the toilet itself… it also minimises your risk … (PP6*,* 30*,* Female)*.

*Also*,* with the measuring tape I’ve cut my fingers so many times. When you’re stretching and pulling back the tape you easily can cut yourself. (PP6*,* 30*,* Female)*

There were also perceived benefits for the patient when using PilOT-Measure’s remote measurement feature. For example, being able to take the popliteal height measurement without having to touch the patient was seen as being a significant potential benefit for patients.

*For example*,* when I’m doing the measurement on the bed… I kind of need to touch you to an extent*,* but if you are using a digital tool*,* you can just zoom into that area and place a point… you don’t need to touch the person and some people don’t like being touched necessarily… (PP6*,* 30*,* Female)*.

#### Effort expectancy

All Participants reported that they were satisfied with the ease of use of PilOT-Measure and found the minimalist user interface made the task of measuring items an intuitive one. All reported that they were able to place markers and use the application for its intended purpose. Some suggested that perhaps their familiarity with touchscreen devices may have helped to reduce any learning overhead. However, some participants did state that they experienced some difficulty when placing or locating the initial measurement point.

*I was impressed…it was super easy I’m not very technologically inclined*,* so I was grateful for its simplicity. (PP11*,* 41*,* Female)*

*I think it’s pretty friendly*,* I think the thing is that because it’s a tablet and I’m used to kind of tapping and using a phone anyway*,* that it’s quite an easy link to make. The thing that I found most difficult was locating the point that I want to establish the measurement from with my finger … I wonder whether using a stylus would improve its accuracy…. (PP3*,* 37*,* Female)*

More specific issues relating to placing the measurement points included difficulty in establishing whether the point placed was truly adjacent to the item’s edge.

*I like that you get to do it yourself but sometimes I question whether it has actually got the exact true edge of the object that I’m trying to measure. I have a hard time making sure on whether it was the true edge and that part made me a little bit worrisome. (PP11*,* 41*,* Female)*

It was also stated that there was some difficulty in placing the measurement marker on shiny surfaces, but it was noted that a change in their physical location and the point-of-view of the device corrected this issue.

*Think it’s pretty self-explanatory and pretty straightforward. I do think some things need smoothing out such as placing the initial dot on shiny surfaces such as the bath… But otherwise*, everything else was simply *bam-bam* and the dots appeared and measure it instantly. (PP8,* 26*,* Female)*

Some participants suggested how PilOT-Measure could be improved to overcome the measurement marker placement issue. For example, one suggestion was to overlay augmented hard-line edges onto the edge of the item on-screen to help the user understand and visualise where the edges of items are.

*If you were to put the camera up and it could identify hard edges and give you a track or tracer feature…where you can see that the tracer is showing a projected line [onto each edge]. (PP4*,* 26*,* Male)*

#### Social influence

Participants felt that the depth sensing technologies that PilOT-Measure deploys, and the features and functionality that it delivers, raises important questions about the potential for automation of the FRA process and the possibility of patient self-assessment. It also prompted discussion about potential barriers and facilitators to adoption of such applications in practice. User age and experience were suggested as factors that can impact the adoption of PilOT-Measure. However, it was believed that if academic research showed that there are clear benefits to using the application, then ultimately, this would over-ride these potential barriers to acceptance and practitioners may be more willing to use the application in practice.

*I think because it’s a new item… it will always be met with sheer reluctant force… but that’s the normal human way to see this as a challenge potentially… but I think once the researched is accessed… for example if I don’t see the research behind a new application I don’t necessarily buy it… once that standard is met for everyone across the board I think it will be fine (PP7*,* 29*,* Female)*.

*Granted*,* as a practitioner*,* and as part of our code of ethics is to question and make sure findings to be true for ourselves as well … and so if there is proven information out there that we can access*,* the scholarly journals or research and studies that we find it to be valid and reliable tool then I would definitely be keen and happy to use it. (PP11*,* 41*,* Female)*

#### Augmenting equipment provision

It was felt that PilOT-Measure has great potential as a tool that enables enhanced patient-practitioner collaboration. One participant suggested using the digital images of the patient’s home captured by PilOT-Measure as a visual aid to focus patient-practitioner discussion around possible assistive equipment fitment and possible adaptations that could be made to the patient’s home. Ultimately, such discussions could facilitate joint decisions being made about home adaptations and potentially help reduce levels of assistive equipment abandonment.

*Health care professionals could use models and use these to explain to the client… and show them this is where I’m putting a railing in your bath … and this is what it looks like. This would help with us explaining why and potentially lead to the conversation of taking the clients approach instead (PP9*,* 36*,* Male)*.

Furthermore, one participant suggested that additional features could be built into PilOT-Measure to help visualise what the home adaptations may look like once made, again helping to encourage shared decisions about potential adaptations.

*I think you could use it from a collaborative approach … what would be really good is to put an overlay of all these equipment options to see what it would look like… if you could drag and drop and show them what it looks like [in their home] it might help them make that decision and be much more patient centred than just prescribing a whole bunch of stuff that they are never going to use because they think it’s ugly. (PP4*,* 26*,* Male)*

#### Clinical Self-Assessment

There was general consensus between participants on the potential value of using PilOT-Measure as a tool that can enable some patients to carry out FRAs themselves, instead of requiring an OT to visit the patient’s home. It was felt that if PilOT-Measure could be used by patients, or by carers or members of their family, to carry out FRA self-assessments. There could be significant benefits in terms of time saving for OTs but also for the patient in terms of patient-empowerment and the significant benefits this carries.

*Patient-empowerment is a huge part of OT and if we can get the patient to the point where they are confident enough [to use PilOT-Measure] or their loved ones can… doing the measurement will only benefit them… and it will also benefit us*,* it saves time from having to do it ourselves… the only slippery slope is how accurate is their measurement? … but if you can take images of their measurement and cross reference this for validity purposes then it should be fine … (PP7*,* 29*,* Male)*.

*I would say that there probably will be some people that wouldn’t be capable of using it*, i.e.,* those that aren’t familiar with these kinds of technologies*,* however there’s an awful lot of people that are*,* such as family members and would still be very useful to free OT time for more cost-effective tasks. (PP4*,* 26*,* Male)*

One participant was concerned, though, about the potential for measurement errors in self-assessments and stressed the potential complexity of the task of taking measurements, given that environmental factors must also be taken into account when measuring.

*I would still think it’s possible*,* if they know what they are doing*,* sometimes they do the measurements for themselves … but they might alter the results to gain access to equipment … it will increase the risk of an accident if they don’t do it properly. Just doing the measurement is possible*,* however*,* having the user to consider everything around them [environment] and how they use it isn’t realistic. (PP6*,* 30*,* Female)*

#### Privacy

Privacy concerns were a common factor raised by participants due to PilOT-Measure’s usage of camera technologies and recording of digital images of the patient’s home. Participants tended to think that although privacy is likely to be raised as a concern by patients and practitioners, in time and with the correct training, privacy concerns could be managed and overcome.

*They may feel like their privacy is being violated they may feel like*,* oh you’re taking pictures. (PP8*,* 35*,* Female)*

*I would say that individuals would be hesitant at first but given enough training I’m sure they’ll [see the value]…and again the worry usually comes when new processes are enforced but not much information is given to support the change in practice… for example when taking pictures of equipment placement at a clients home… we are now required to bring up the conversation of privacy and ensure they can’t be identified if the case is transferred to a different unit… sometimes clients don’t even think about it and mentioning it can trigger their self-awareness. (PP17*,* 39*,* Female)*

## Discussion

PilOT-Measure, a depth-perception enabled mobile application for carrying out fully digitised falls risk assessment, has been presented in this study. The performance of the application was evaluated via a user-based study involving 21 participants conducted within an Assisted Daily Living Suite (ADL). The evaluation explored how effectively (accuracy, and accuracy consistency) and efficiently (task completion time) measurements can be taken and recorded by PilOT-Measure compared with a handheld tape-measure and paper-based guidance booklet. Furthermore, usability measures (SUS) and post-task interviews were conducted to investigate comparative user satisfaction, and to explore what the perceived challenges and opportunities of the use of PilOT-Measure are for use in clinical practice.

The first research question explored the accuracy of measurements recorded using PilOT-Measure and the tape measure and booklet. The results show, that in absolute terms, PilOT-Measure outperformed the booklet by producing a smaller median error difference in six out of 11 cases. The one-sampled Wilcoxon Signed Rank test comparison against true measurement values indicate that, in most cases (eight out of 11), PilOT-Measure generated measurements that were not statistically different from the true measurement values, hence indicating acceptable levels of measurement accuracy in all of these cases. The tape measure and booklet performed almost as well, with seven out of 11 measurements being accurate enough to be not significantly different from the true measurement values. Bath External Width, Chair Depth, and Chair Width were accurate for both PilOT-Measure and the booklet. However, when looking at absolute values, PilOT-Measure produced smaller median error differences for both Bath External Width and Chair Depth. A notable difference was that PilOT-Measure produced accurate measurements for both Toilet Height A and B, compared with the booklet which did not achieve accurate measures for these items. The fact that PilOT-Measure produced a full set of accurate measurements for the toilet and chair is a promising and important outcome as research has indicated that toilet or chair raisers are the most commonly administered pieces of assistive equipment within the home setting [75]. Therefore, accurate measurements of these items is crucial to ensure safe transfer on and off the toilet and chair and can be an impeding fall risk factor if the correct height isn’t acquired during the falls risk assessment [76, 77]. One noticeable observation is that all items that PilOT-Measure achieved significantly more accurate measurements for (Bath Height, Chair Height, Toilet Height A, Toilet Height B) had higher median error difference values compared with the booklet. This suggests that the observed PilOT-Measure error differences may have been more consistently inaccurate than the booklet, hence enabling the median error difference to be a higher value, yet still not being significantly different from the true value overall. One possible explanation for a consistent inaccuracy overhead may be related to the measurement marker challenges that participants stated they experienced when using PilOT-Measure, particularly finding it difficult to place markers precisely on a surface edge. Overall, in terms of measurement accuracy, PilOT-Measure and the booklet performed at comparable levels, with PilOT-Measure only achieving one more significantly accurate measurement item than the booklet. This indicates that PilOT-Measure appears to be capable of achieving measurement accuracy at a similar level to the current state of the art. Improvements would need to be made to PilOT-Measure in order for it to clearly outperform the booklet.

The second research question compared the relative accuracy consistency of the two measurement guidance tools. The results revealed that, when considering absolute median error differences, the measurements recorded in the booklet significantly outperformed PilOT-Measure in six of the 11 cases. Although the differences in the remaining five cases are not statistically significant, three cases lead to the booklet generating larger error differences, all of which were chair measurement items. All five cases generated error differences that were less than one centimetre with effect sizes varying between medium and trivial. In practical terms, some research has suggested that acceptable margins of error within the pre-assessment visits and identified a 1 cm to 5.8 cm difference to be within acceptable criteria [13]. Nevertheless, overall, despite the absolute error values being relatively small, the booklet recorded more consistently accurate measurements compared with PilOT-Measure. There is a need to explore how PilOT-Measure performance can be improved in terms of accuracy consistency. One possible explanation for PilOT-Measure’s less consistent accuracy may be related to the challenges participants stated they experienced when attempting to place measurement markers close to the edges of items on-screen, as inaccurate placement of measurement markers would lead to consistently inaccurate measurement. There is a need to explore how measurement marker placement can be optimised so that overall measurement accuracy and accuracy consistency can be improved upon. This would be an important function to focus and deliver on, as currently accuracy consistency is not on par with the booklet.

The third research question evaluated the task completion times for PilOT-Measure and the booklet in terms of individual measurement tasks for each item respectively. The results revealed that PilOT-Measure enabled participants to capture individual measurement items significantly faster in 6 out of 11 cases when compared to those recorded in the booklet. In two out of 11 cases (Bath External width and Toilet Height B: Floor-Seat), participants were able to capture measurements more efficiently with the tape measure and booklet. For the remaining three items there was no significant difference, between PilOT-Measure and the booklet, in the time taken to measure items. Considering the current time-demands associated with pre-assessment visits [78] and the administrative overhead that frequently follows in the form of transcribing interview data, transferring paper measurement results and interdepartmental review and communication efforts [79], a clear benefit is identified in terms of productivity in favour of PilOT-Measure. Existing research has shown support for this notion suggesting that ICT in Occupational Therapy Home Assessments offer a valuable potential to improve service delivery and efficiency, though further work is required to identify it’s superiority in terms of patient-outcomes [80–84]. These results are promising and indicate that digital depth-enabled falls risk assessment applications may present valuable resource saving alternatives to current paper-based practices.

The fourth research question evaluated the usability of the two measurement guidance tools by comparing participant responses to the Systems Usability Scale (SUS). The results revealed that PilOT-Measure achieved a higher overall SUS score versus the tape measure and booklet (76.0 vs. 58.5 respectively). In all cases, PilOT-Measure delivered positive gap scores which indicate that, for all 10 SUS items, participants tended to be more positive about the application compared with the booklet. In statistical significance terms, six out of the 10 SUS items (S1-S4, S8 and S10) resulted in a significant difference all in favour of PilOT-Measure. Participants were positive about PilOT-Measure said it delivered a significantly improved user experience in terms of both the usability and learnability constructs. Individual SUS item results reveal that participants had a significant positive preference for PilOT-Measure in terms of frequency of use (S1), being significantly less complex (S2), easier to use (S3), not requiring support (S4), less awkward to use (S8) requiring a lower learning overhead (S10). These results are encouraging particularly in light of the on-going resource constraints in the healthcare sector and the need to integrate a wider range of novel technologies that help to automate and optimise efficiency of practice [85].

The fifth research question investigated clinicians’ views of PilOT-Measure and the perceived challenges, opportunities and intention to adopt the measurement tool in practice. In terms of Performance Expectancy, participants suggested that PilOT-Measure has the potential to improve the efficiency of taking measurements and notes about a falls risk assessment visit, potentially reducing the required time and administrative overhead of having to keep paper records of home visits. It was also suggested that measurement accuracy could be improved if PilOT-Measure better enabled practitioners to refer to the latest measurement guidelines whilst carrying out FRAs. Having a single shareable digital record of the FRA was also said to provide the potential to enhanced inter-professional joint decision making, which is in line with existing Tele-OT research [86–88] and health technology-based research that explores the benefits of applying visualisation technologies in paper-based assessment practices [28, 80, 89–92]. In terms of improved health and safety within the workplace, it was reported that use of PilOT-Measure could potentially minimise several risk factors such as contact with unsanitary toilet surfaces, potential lacerations from using of industry standard metal tape measures, and could remove the need to touch the patient when taking some measurements, as some patients do not wish to come in contact with others as part of the FRA [93], especially in light of the recent COVID-19 pandemic.

In terms of Effort Expectancy, participants were satisfied with the ease of use of PilOT-Measure and found the minimalist user interface an intuitive one. Although all participants were able to place measurement markers and use the application, some did state that they had some difficultly establishing whether the first measurement marker was placed correctly within 3D space and precisely on the desired surface edge. Furthermore, some participants also noted that placing markers on reflective surfaces such as the Bath, Toilet, which caused further issues. This qualitative finding corresponds with our quantitative statistical observation that PilOT-Measure median error differences for accuracy consistency was higher for both bath and toilet compared with the booklet. This highlights a need to explore how marker placement can be improved for shiny surface [94–96].

Factors relating to Social Influence and Clinical Self-Assessment themes, included the suggestion that PilOT-Measure could serve as a tool that enables patient self-assessment practice, hence, potentially bringing cost savings by reducing the time and resource overhead placed on the health service and practitioner [97, 98]. It was suggested that the patient’s ability to use and carry out self-assessments using PilOT-Measure may be dependent on factors such as age and experience and that accuracy of measurements may be of concern if patients were not able to use the application effectively.

Augmenting Equipment Provision highlighted the potential for using PilOT-Measure to help facilitate the joint-decision making process between the patient and practitioner. Digital images of the patient’s home recorded using PilOT-Measure could be used as a visual aid that could help patients to visualise what possible adaptations may look like within the home, hence, helping joint-decision making. This potential area of application is encouraging and seems consistent with existing research which investigates the benefits depth-perception visualisation applications within clinical settings [39, 99–101].

Finally, Privacy was noted as a point for consideration when using PilOT-Measure, and in particular, the use of image recording equipment within a patient’s home and that patients may be concerned with how these images may be used in the future. Patient privacy, and dealing with patient concerns about privacy, is an important topic that must be carefully considered when deploying such technologies within patient homes. The use of sensor technologies within healthcare and being aware of the concerns that patients may have about the use of such technologies is an ongoing area of research and it is important that appropriate training is given to practitioners to ensure that data privacy maintained and patient queries about privacy are dealt with in a sensitive and informative way at all times [102, 103].

## Conclusions

PilOT-Measure, a mobile depth-sensing measurement application for carrying out falls risk assessments, has been presented in this study. Based on the key findings, future work will primarily focus on exploring how measurement accuracy and accuracy consistency can be improved using PilOT-Measure and mobile depth-sensing technologies. Although PilOT-Measure performs marginally better than the booklet in term of overall accuracy, there is a need to further explore how accuracy can be improved to deliver performance gains that are well above that of traditional paper-based methods. This coupled with the need to improve marker placement to overcome the sub-optimal performance of PilOT-Measure, in terms of accuracy consistency, poses the most immediate challenge. More specifically, appropriate de-homogenisation techniques pertaining to Translation Rotation and Scaling (TRS) factors will be considered to more aptly interpret and render 2D touch markers to that of 3D point-cloud data [104, 105]. Furthermore, methods such as applying contextual/non-contextual segmentation or edge-detection filters to 2D images can assist in initial marker selection [106]. The generation of 3D depth-maps by means of organised point-cloud data sets (i.e., RGB-D) has also shown great potential in mapping the 2D and 3D perspective geometry cues [107–109], and will be further explored in future research.

Other areas of future research could include exploration of the perceived privacy issues, from a patient’s perspective, that arise from using depth sensing technologies as part of the FRA process. Whilst there have been studies that explore privacy issues that relate to the use of depth sensing technologies within home settings [102], older-adult’s privacy considerations [103], and privacy recognition technologies for daily-living activities through RFID sensors [110], there does not appear to be any research relating to the use of such technologies within the falls risk assessment process. There is a need to establish whether an application like PilOT-Measure could feasibly be used by patients to enable them to carry out FRA self-assessments and establish the clinical utility of using an application like PilOT-Measure for this purpose.

## Supplementary Information

Below is the link to the electronic supplementary material.

Supplementary Material 1

## Data Availability

The datasets used and/or analysed during the current study are available from the corresponding author on reasonable request.
