# Supplementary material for: PilOT-Measure: a mobile 3D depth sensing application to support accurate and efficient clinician-led home-based falls risk assessments
Source: BMC Med Inform Decis Mak. 2025 Sep 24;25:332. doi: 10.1186/s12911-025-03149-7 (PMC12462305; doi:10.1186/s12911-025-03149-7)
Supplement: Supplementary file 1 — Supplementary Material 1 [file 12911_2025_3149_MOESM1_ESM.pdf]

Semi-Structured Interview Questions

No.	Question
1	What is your opinion of the PilOT-Measure system?
2	What is your opinion on the Paper-based guidance leaflet?
3	How do you feel when measuring indoor furniture items using either systems?
4	Do you think there is any observed improvement between the two systems, if so what?
5	Would you be able to use either of these systems without professional input?
6	Do you think there are there any negative aspects of either systems?
7	Would you like to have this system at home – if so which one would you prefer?
8	Do you think mistakes can be made easily using either system?
9	Do you think either system would interfere with your daily routine?

Further probing questions

Perceived Usefulness

1. How do you think The PilOT-Measure System will affect your ability to perform your job (i.e occupational therapy measurement based activities) **

    a. How effective do you think your time spend in the PilOT-Measure System will be?
    b. In what way will this affect your other duties?
    c. In what way do you think the PilOT-Measure System will improve or worsen your day-to-day activities

Perceived Ease of Use

1. Tell us how user friendly you think The PilOT-Measure System is?
    a. How do you think the user interface in The PilOT-Measure System will work with general measurement
    b. How do you think it will be for you to do what you're doing in The PilOT-Measure System – will it be better over time?

Subjective Norm

1. Tell us what you think about The PilOT-Measure System without having any direct experience of it?
    a. Who affects this view?
2. Do you think you can be influenced by other people's opinions about The PilOT-Measure System?
3. Tell us how you think the view of an OT care unit will be affected the introduction of The PilOT-Measure System

Education / Training

1. Have you had any indoor furniture measurement training using the standardized practices under the college of Occupational Therapy in your course?

Experience

1. Tell us about your previous experience with furniture measurements in Occupational Therapy
2. Has your vision of the PilOT-Measure systems changed your knowledge about the current measurement practices in OT?
3. How much influence do you feel you have on the development of the PilOT-Measure System for furniture measurement as part of the Fall prevention processes
4. Would you like to have had more influence?
    - (If yes) In what respect?

Job Relevance

1. Tell us how important The PilOT-Measure System will be for your work experience
2. How well prepared do you feel about working with the PilOT-Measure system in real life settings?

Depending on whether the answer is positive or negative to the above question

- (Positive) What caused you to feel comfortable?
- (Negative) What would make you feel better prepared?

3. How do you think your work performance and assessment output will be affected or changed due to The PilOT-Measure System

Output Quality

1. Tell us how you think patient safety will be affected by The PilOT-Measure System
2. Do you think the PilOT-Measure system provides enough environmental data/information that can be used to make a valid and concise decision on home adaptations?
3. Do you think this system can assist with the home adaptation process if the data captured and items measured were sent to you via a digital platform

Result Demonstrability

1. Do you think this system would function to perform as a self-assessment tool/guide for the elderly patients and what would need to be improved to achieve this?
2. How would you envision this system interacting with OTs working alongside patients when performing home adaptations?
